# Machine Learning–Driven Surrogate Modeling and Operating-Point Selection for a Microfluidic Diffusion-Membrane Platform for Transdermal Drug Delivery

**DOI:** 10.3390/membranes16070239

**Published:** 2026-07-15

**Authors:** Tara Torabi, Mahsa Jafar Harasy, Jafar Tahmoresnezhad, Samira Malekmohammadi, Adolfo Iulianelli, Kamran Ghasemzadeh

**Affiliations:** 1 Chemical Engineering Department, Urmia University of Technology, Urmia 5716617165, Iran; taratorabi79@gmail.com; 2 Computer Engineering Department, Urmia University, Urmia 5716617165, Iran; mahsajafrhrsi@gmail.com; 3 Faculty of Information Technology and Computer Engineering, Urmia University of Technology, Urmia 5716617165, Iran; tahmores@gmail.com; 4 Henry Royce Institute, University of Manchester, Manchester M13 9PL, UK; samira.malekmohammadi@manchester.ac.uk; 5 Department of Materials, School of Natural Sciences, University of Manchester, Manchester M13 9PL, UK; 6 Institute of Membrane Technology of the National Research Council (CNR-ITM), Via P. Bucci 17C, 87036 Rende, Italy; 7 School of Engineering, University of Edinburgh, Edinburgh EH9 3JW, UK

**Keywords:** transdermal drug delivery, membrane technology, machine learning, microfluidic diffusion

## Abstract

Microfluidic diffusion systems provide a powerful in vitro platform for evaluating transdermal drug delivery (TDD), yet their predictive capability is often constrained by limited experimental datasets and nonlinear transport behavior across membrane—device configurations. This study integrates machine learning (ML) with microfluidic experimentation to develop accurate and generalizable surrogate models for cumulative drug permeation under different hydrodynamic and membrane conditions. This work presents an ML-augmented microfluidic TDD framework for predicting cumulative drug permeation from small experimental datasets. Caffeine cream permeation was examined across twelve device—membrane configurations (sMDC, mMDC, and LiveBox2 paired with PET, CA, rat skin, and alginate) at three perfusion flow rates. For each configuration, SVR, MLP, RFR, GBR, XGB, and KNN models were trained and cross-validated using only 33 experimental measurements. SVR showed the strongest overall performance among the evaluated models, achieving test R^2^ values typically above 0.97 and RMSE values of <1–3 µg/cm^2^, accurately capturing the nonlinear time-flow-cumulative mass behavior of TDD profiles. A domain-bounded Gaussian-noise augmentation strategy was used to increase local sampling density while keeping augmented values within the experimentally observed time and cumulative-mass ranges. Polynomial equations were obtained from the predictions of SVR to capture the interaction between inputs and outputs. The trained SVR surrogates were then used for automated steady-state identification and surrogate-based operating-point selection, revealing the dependence of the selected flow rate on membrane permeability and device geometry. Alginate consistently delivered the highest steady-state cumulative mass across all systems (up to ~448 µg/cm^2^), establishing it as the most efficient TDD membrane among those evaluated. Finally, compact third-degree polynomial equations were derived from the SVR predictions, enabling explicit analytical prediction and rapid design-space exploration. Overall, these ML-derived models and analytical equations provide a fast, low-cost tool for predictive design, enabling rapid microfluidic system evaluation and operating-condition selection, and significantly accelerating the development and screening of next-generation TDD platforms.

## 1. Introduction

Drug delivery strategies are crucial for turning therapeutic compounds into effective treatments. A significant issue with systemic drug administration is the uncontrolled distribution of drugs throughout the body, which can lead to degradation in healthy tissues and necessitate higher doses to reach therapeutic levels at the target site. To tackle these problems, modern delivery methods focus on targeting therapeutics accurately while lowering dosage needs and side effects. Recently, new drug delivery technologies have gained attention for their ability to improve effectiveness, safety, and precise delivery performance [1,2].

Among these technologies, transdermal drug delivery systems (TDDSs) have become a particularly appealing alternative due to their non-invasive, patient-friendly design and capability for controlled drug release. TDDSs, commonly referred to as patches, deliver drugs through the skin into systemic circulation, bypassing gastrointestinal metabolism and reducing dosing frequency compared with oral administration. Their painless, sustained-release profile offers improved patient compliance, making them suitable for chronic and long-term therapies. Modern TDDS are typically designed as drug-in-adhesive systems, in which the active pharmaceutical ingredient is directly dispersed within a pressure-sensitive adhesive layer supported by a backing membrane. This configuration simplifies manufacturing and reduces cost compared with earlier reservoir-type systems, which consist of a drug reservoir separated from the skin by a rate-controlling membrane. Although reservoir—membrane systems were widely used in early TDDS designs, they are now less common in commercial products. In both configurations, additional components such as adhesives, backing layers, and penetration enhancers can be incorporated to regulate drug transport and maintain therapeutic concentrations over extended periods [3,4,5,6].

Since TDDSs directly impact human health, they must undergo thorough preclinical testing to assess safety and efficacy before approval for public use. Traditionally, this process relies heavily on animal experimentation, which is costly, time-consuming, ethically contentious, and often fails to accurately predict human physiological responses. Similarly, conventional two-dimensional cell culture models present limitations, as cells behave differently in these simplified environments compared to their native, three-dimensional contexts within the human body. Organ-on-a-chip technology has emerged as a promising alternative that addresses many of the shortcomings associated with both animal testing and traditional in vitro models. These microfluidic-based systems are engineered to replicate the structural, functional, and mechanical features of human organs, allowing for dynamic simulation of physiological activities and cellular responses. By replicating the physical and chemical microenvironment, organ-on-a-chip systems can better preserve cellular morphology and functionality, enabling more accurate modeling of organ-level responses. To date, organ-on-a-chip devices have successfully mimicked the functions of key organs such as the lung, liver, kidney, and intestine. By recreating key aspects of tissue architecture and fluidic flow, organ-on-a-chip devices enable direct observation of biological behavior rather than relying on extrapolated data from animal models. These chips are compact, cost-effective, and require minimal sample volumes, making them highly versatile tools for biomedical research and drug development. With continued refinement and validation, this technology has the potential to accelerate drug approval timelines, expand the scope of therapeutic testing, and substantially reduce or even replace the need for animal experimentation [7,8,9]. Transdermal patches can be integrated on organ-on-chip devices for effective assessment of physicochemical characteristics, in vitro drug release profiles, skin permeation behavior, and adhesive performance [4].

Despite the advantages of microfluidic platforms for studying transdermal delivery, quantitative characterization of drug diffusion in these systems remains challenging. Experimental datasets from TDDSs and microfluidics usually have limited temporal sampling because of practical constraints on how often samples can be collected. This issue is commonly noted in microfluidic diffusion research [10]. Drug transport is further influenced by nonlinear and geometry-dependent interactions between membrane permeability, shear-dependent mass transfer, and channel architecture, making cumulative mass profiles highly sensitive to device design and flow regime [11,12]. Moreover, each diffusion experiment is experimentally costly and labor-intensive, especially when biological barriers or multilayered constructs are used, restricting the number of feasible replicates and test conditions [13].

These experimental constraints have traditionally been addressed through trial-and-error optimization, where researchers iteratively adjust system parameters to achieve the desired release behavior. While this approach has been adequate for simpler systems, it relies heavily on expertise and becomes increasingly impractical as drug delivery platforms incorporate complex materials and nonlinear transport phenomena. Computational simulations offer another path, but techniques capable of capturing the detailed structure–function relationships of TDDSs are often computationally demanding, slow to parameterize, and unsuitable for rapid or large-scale predictive screening [14]. Recent advances in artificial intelligence (AI) and machine learning (ML) now offer powerful alternatives by enabling predictive, data-driven modeling of drug–skin interactions, material properties, and release kinetics [14,15]. These methods can screen large parameter spaces, identify optimal formulation components, and accurately forecast drug permeation and deposition within skin layers. By reducing experimental workload and accelerating design cycles, AI/ML support faster and more efficient formulation development. Additionally, AI-assisted image analysis can inform early decisions by predicting dermal responses and skin conditions relevant to transdermal delivery. Collectively, these technologies are reshaping TDDS research by enhancing formulation optimization, improving therapeutic performance, and supporting the broader shift toward personalized drug delivery [16].

Although ML-based models have been widely explored across numerous scientific disciplines, their application in TDDSs remains limited, particularly in the context of TDDSs integrated with microfluidic diffusion platforms. Existing studies, such as Kocsis et al. [17], focus mainly on optimizing device geometry and flow behavior without offering quantitative predictive models for drug transport. This lack of data-driven modeling represents a central scientific gap, as current TDDS–microfluidics research relies primarily on empirical diffusion profiles and CFD visualization, with no tools capable of forecasting cumulative mass under new operating conditions. To address this gap, the present study provides a systematic evaluation of multiple ML algorithms for predicting the relationships between time, flow rate, and cumulative mass across diverse membrane—device configurations. Six regression models, including Multilayer Perceptron (MLP), Gradient Boosting Regressor (GBR), Extreme Gradient Boosting (XGB), K-Nearest Neighbors (KNN), Random Forest Regressor (RFR), and Support Vector Regression (SVR), were compared to assess predictive accuracy and generalization behavior. Based on these comparisons, SVR was identified as the most reliable model and was subsequently used to derive explicit predictive equations that capture the nonlinear interactions between inputs and outputs. Furthermore, the trained SVR framework enabled automated steady-state identification and surrogate-based operating-point selection, yielding the selected flow rates, steady-state times, and steady-state cumulative-mass values for each membrane—device system.

## 2. Methodology

### 2.1. Configuration Design of the Microfluidic Diffusion Systems

In this study, the configuration of the microfluidic diffusion systems reported by Kocsis et al. [17] was adopted as the experimental reference architecture. These systems were specifically designed to provide a controlled microenvironment for quantifying molecular transport under well-defined flow, geometric, and membrane conditions.

To investigate the permeability of a model topical formulation (caffeine cream), three microfluidic device types, including (1) single-channel microfluidic diffusion chamber (sMDC), (2) multichannel microfluidic diffusion chamber (mMDC), and (3) LiveBox2 system, were evaluated in combination with four membrane materials: polyester (PET), cellulose acetate (CA), rat skin, and alginate scaffold. This resulted in twelve unique device–membrane configurations. Diffusion surface area and membrane thickness values are presented in Table 1.

Across all device types, the fundamental geometry involved parallel donor and receiver microchannels separated by a semi-permeable membrane. In the sMDC and LiveBox2 systems, two channels were positioned on opposite sides of the membrane, while the mMDC incorporated multiple parallel receiver channels to increase the membrane contact area. The membrane was positioned centrally on the donor channels, enabling solute transport from the donor compartment (containing the caffeine formulation) to the receiver stream.

Caffeine was selected as a model permeant, because it is widely used in topical and transdermal permeation studies and has well-characterized skin-transport behavior. Its relatively low molecular weight (194.19 g/mol), hydrophilic character, analytical detectability, and established use in dermal absorption studies make it suitable for comparing diffusion behavior across microfluidic membrane configurations. However, caffeine does not represent all transdermal active compounds [18].

**Figure 1:**
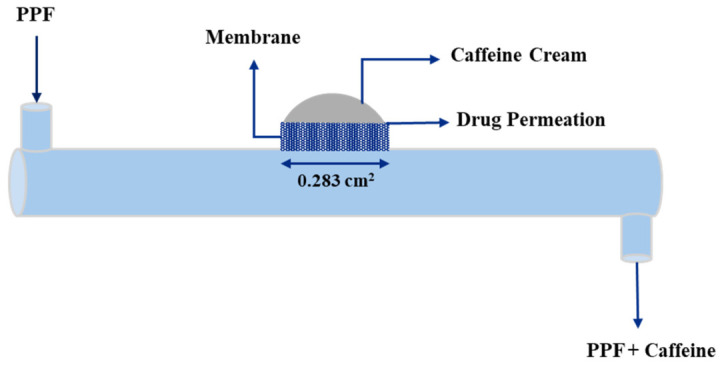
Schematic of the sMDC system.

A peripheral perfusion fluid (PPF) was supplied to the receiver inlet to collect the permeated solute and maintain a constant convective driving force along the channel. Consequently, the receiver outlet contained a mixture of the PPF and diffused caffeine. Figure 1 illustrates the schematic configuration of the sMDC system.

The modularity of these devices allows systematic variation of residence time, membrane contact area, and mass-transfer path length, enabling controlled exploration of different diffusion—convection regimes. To evaluate the influence of geometry and membrane type on transport behavior, diffusion experiments were performed for each of the 12 configurations at three PPF flow rates (4, 40, and 100 μL/min).

For every experiment, the donor channel received the caffeine formulation, while the receiver channel contained the PPF. Time-dependent cumulative masses at the receiver outlet were recorded and used to construct diffusion profiles (cumulative mass vs. time). Therefore, each diffusion experiment was performed in triplicate, providing three independent cumulative-mass profiles per device—membrane—flow combination. Cumulative-mass measurements were recorded at regular 30 min intervals over a 300 min duration, producing time-resolved diffusion profiles with sufficient resolution for downstream ML modeling. From these replicates, averaged cumulative-mass curves were generated, and 33 representative datapoints were extracted per configuration for ML training. Table 2 presents the controlled, measured, and derived parameters in TDDS—microfluidics experiments.

In the dataset used for ML model development, each membrane—device configuration contained 33 time-resolved datapoints, corresponding to three PPF flow rates and 11 sampling time points per flow rate. Since twelve membrane—device configurations were evaluated, the total dataset used for modeling consisted of 396 datapoints. The dataset was based on digitized cumulative-mass profiles and did not include raw replicate-level measurements or datapoint-specific standard deviations. Therefore, replicate-level experimental uncertainty could not be directly calculated or propagated into the ML models. Accordingly, the model-performance errors reported in this study should be interpreted as deviations from the available digitized cumulative-mass profiles, rather than as direct estimates of the full experimental variability of the original measurements.

### 2.2. Integration of Machine Learning and Surrogate-Based Operating-Point Selection

Predicting cumulative mass in TDDS microfluidic systems is inherently challenging, because drug transport depends on nonlinear interactions among membrane properties, microchannel geometry, flow-dependent mass transfer, and time-varying concentration gradients relationships that are difficult to capture from sparse experimental datasets.

Conventional mechanistic or CFD-based models can in principle simulate these dynamics but are often cumbersome: they require detailed knowledge of membrane permeability, porosity, and diffusion coefficients; involve computationally expensive numerical solvers; and must be reparameterized for each new membrane–device configuration, making them impractical for rapid predictive screening. Moreover, existing microfluidic diffusion datasets, such as those of Kocsis et al. [17], provide only discrete experimental cumulative-mass profiles rather than continuous functional relationships, creating a need for surrogate models capable of interpolating and extrapolating beyond the limited sampling regime.

Motivated by these challenges, all modeling was performed using a custom Python 3.13 workflow designed to process the experimental TDDS microfluidics dataset [17] and train an independent predictive model for each microfluidic device–membrane configuration. The dataset included two input variables, including time (0–300 min) and PPF flow rate (4, 40, and 100 µL/min), together with the measured response (cumulative mass). For each of the twelve device–membrane combinations, a subset of 33 datapoints was extracted and analyzed separately, ensuring that each model was trained exclusively on the data associated with its respective configuration without cross-contamination between designs. Initially, six ML models, including MLP, GBR, XGB, KNN, RFR, and SVR, were trained solely on the original 33 experimental points for each configuration to compare their reliability, accuracy, and generalization performance. For each device—membrane configuration, the data were randomly divided into an 80% training subset and a 20% held-out test subset using a fixed random seed. A chronological time-series split was not applied, because the aim of the model was to interpolate cumulative permeation within the experimentally investigated time-flow domain rather than to forecast future time points beyond the measured experimental window and extrapolation to untested conditions. When a valid replicate or curve identifier was available, the split was performed using a group-based strategy so that related observations remained within the same subset. Otherwise, a randomized point-level split was used. The same splitting logic was applied consistently across configurations.

These six models were selected based on their diverse representational capabilities. The MLP represents a class of artificial neural networks capable of capturing complex nonlinear and multivariate relationships [19,20,21,22]. SVR is a nonlinear regression method noted for its robustness and strong predictive performance when working with small or limited datasets [23,24]. RFR integrates multiple decision trees through ensemble learning, improving predictive accuracy and model stability [25]. GBR is another powerful ensemble technique that iteratively refines weak learners to achieve high predictive performance across a wide range of regression tasks [26]. XGB extends this principle using an optimized and computationally efficient implementation of gradient boosting, offering superior scalability and speed [27,28]. Finally, KNN is a simple, nonparametric approach that generates predictions based on similarity between feature observations [29].

All these models used the same two input features (time and flow rate) and predicted cumulative mass. Feature preprocessing was implemented using a scikit-learn pipeline to guarantee consistent handling across training, cross-validation (CV), and testing. Missing values were imputed using the median of the training subset, after which both features were normalized to the [0–1] range using MinMaxScaler, fitted strictly on the training data to avoid information leakage. The target variable was also scaled to the [0–1] interval using MinMaxScaler.

Hyperparameter tuning for all ML models was performed independently for each configuration using RandomizedSearchCV with CV inside the training subset. Therefore, hyperparameter optimization was performed only within the training subset using CV, and the selected model was then evaluated on the held-out subset. For each ML model, 40 random hyperparameter draws were evaluated over predefined parameter ranges (summarized in Table S1). These ranges included regularization and kernel parameters for SVR, learning-rate and tree-depth ranges for GBR and XGB, network width and regularization coefficients for MLP, neighborhood size for KNN, and tree-based ensemble parameters for RFR.

A 5-fold CV scheme was used whenever sample availability permitted; when the dataset was too small to support five folds, the number of splits was reduced to ensure a minimum number of samples per fold. This strategy balanced variance estimation with the need to avoid overfitting in extremely limited data regimes. CV folds were designed to prevent temporal leakage, ensuring that all time-series points from a given experimental curve remained within the same fold. The best model for each device–membrane configuration was selected by minimizing CV root mean squared error (RMSE) while simultaneously verifying consistency in R^2^ across folds. The best-performing hyperparameter set for each model was refitted on the full training subset, and model performance was subsequently evaluated on the untouched test data.

Based on these comparisons, SVR was selected for further analysis due to its superior performance on small datasets and its ability to generate smooth, empirical interpolations within the experimentally observed time-flow domain. SVR predictions were first used to generate continuous cumulative-mass curves at the three experimental flow rates for visual and quantitative comparison with the experimental data. A key constraint of the experimental dataset was that each configuration contained only 33 datapoints, reflecting the practical limitations of microfluidic diffusion experiments. This extremely small sample regime poses a substantial modeling challenge: it restricts the ability to characterize nonlinear transport behavior, increases the risk of overfitting and severely limits the applicability of high-capacity models. However, similar limited-data conditions are common in experimental scientific studies where collecting large datasets is impractical due to the cost and complexity of laboratory measurements. ML models have therefore been successfully applied to small experimental datasets, often combined with augmentation or domain-knowledge-guided modeling strategies to improve predictive performance [30,31,32,33]. Therefore, the severe data scarcity in each configuration guided the choice of small-data-compatible algorithms and motivated the use of domain-bounded Gaussian-noise augmentation to improve local sampling density and model stability. To increase the effective sample size while preserving the intrinsic shape of each permeation curve, Gaussian-noise augmentation was applied [34,35,36,37,38]. After the original dataset was divided into training and testing subsets, Gaussian-noise augmentation was applied only to the training data. The test set was kept unchanged and contained only original experimental datapoints. For each configuration, training datapoints were grouped by flow rate so that augmentation was performed independently within each flow condition. For a given flow rate, the corresponding time and cumulative-mass vectors were extracted, and their standard deviations and minimum/maximum bounds were computed. The original training datapoints were then replicated five times. In each replicate, Gaussian perturbations with zero mean were added to the time and cumulative-mass values. The augmentation coefficient was set to 0.05; this value represents a relative noise fraction rather than an absolute standard deviation. Specifically, the actual perturbation standard deviations were calculated as 5% of the within-flow standard deviation of each variable: σt = 0.05 × SD(t) for time and σy = 0.05 × SD(y) for cumulative mass. Flow rate values were not perturbed. The perturbed time and cumulative-mass values were clipped to the original minimum and maximum values within each flow-rate group to avoid generating samples outside the experimentally observed domain. Thus, five noisy replicas were generated from the original training datapoints to increase local sampling density while preserving the overall shape of each cumulative permeation profile. This procedure prevented augmented or near-duplicate samples from appearing in both the training and test sets.

Potential overfitting was assessed through K-fold CV by comparing training and validation R^2^ and error metrics, as well as through parity plots and learning curves showing training versus CV performance.

To identify favorable operating conditions, a surrogate-based discrete search was conducted using the trained SVR models. For each device—membrane configuration and flow rate, the SVR-predicted cumulative mass-time profile was analyzed using sliding-window linear regression. The local slope (dC/dt) was compared with a predefined tolerance, and a flow rate was classified as reaching steady state when the slope remained below this threshold for a minimum required duration and the predicted cumulative mass exceeded a predefined fraction of the maximum value within that profile. This procedure represents SVR-assisted steady-state identification and surrogate-based operating-point selection, rather than Bayesian, evolutionary, or gradient-based numerical optimization.

**Figure 2:**
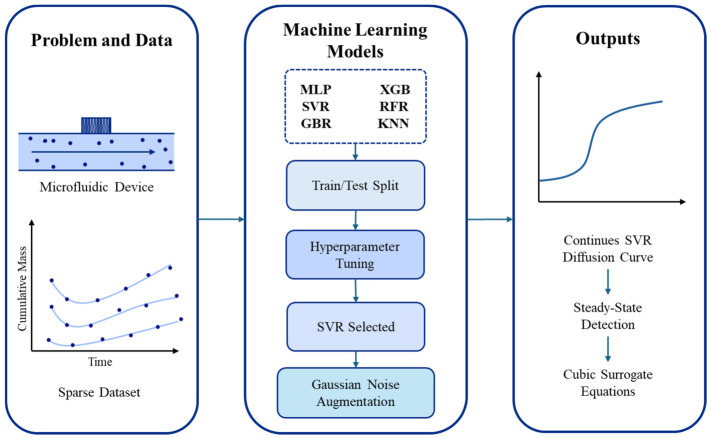
Overview of the TDDS-microfluidic modeling workflow.

Finally, compact analytical surrogate models were developed for each configuration by fitting cubic polynomial regressions to synthetic datasets generated from the SVR predictions. Figure 2 presents an overview of TDDS-microfluidic ML-based modeling workflow.

#### 2.2.1. ML Model Development

In this study, a diverse set of ML algorithms was examined to determine the most effective regression strategy for modeling permeation behavior in membrane–microfluidic diffusion systems.

The evaluated models comprised the MLP, selected for its capacity to learn highly nonlinear patterns; SVR, recognized for its strong performance with limited datasets and robustness in high-dimensional input spaces; the ensemble-based RFR and GBR, both noted for their predictive stability and ability to capture complex feature interactions; XGB, chosen for its strong optimization framework and efficiency on structured datasets; and the KNN algorithm, employed as a straightforward non-parametric benchmark. This combination of models ensures a comprehensive comparison spanning a range of complexities and interpretability levels, all trained under identical data-processing and validation procedures. Detailed descriptions of the MLP, GBR, XGB, KNN, and RFR algorithms are provided in Supplementary Information.

##### SVR Model

**Figure 3:**
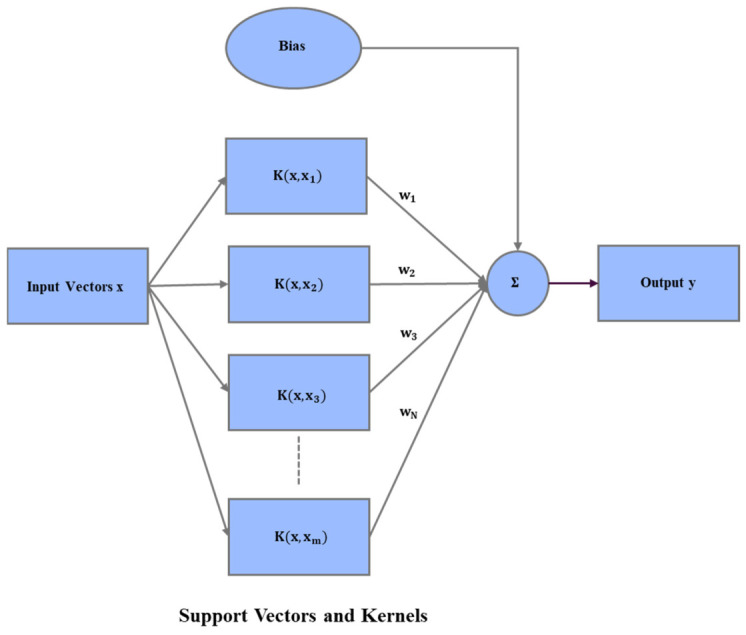
Overall structure of the SVR model.

SVR is well known for its strong predictive performance in situations involving limited datasets, high-dimensional feature spaces, and complex nonlinear relationships between inputs and outputs. In this study, SVR was selected for its robustness and reliable generalization, particularly under conditions where experimental measurements are sparsely sampled and smooth interpolation across operating conditions is required. The overall architecture of the SVR framework implemented in this work is shown in Figure 3 [39,40,41,42].

SVR formulates regression as a convex optimization problem, in which the goal is to minimize a regularized loss function while permitting prediction deviations within an ε-insensitive tolerance band. The learning task can be written as [43]:

(1) $$minimize=\frac{1}{2}{\left\|w\right\|}^{2}+C\sum_{i=1}^{N}\left(\xi_{i}+\xi_{i}^{*}\right)$$

subject to:

$$\left\{\begin{array}{c} y_{i-}w^{T}x-b\leq \xi_{i}+\varepsilon \\ y_{i+}w^{T}x-b\leq \xi_{i}+\varepsilon \\ \xi_{i},\xi_{i}^{*}\;\geq 0 \end{array}\right.$$

where w and b define the regression hyperplane, $\xi_{i}$ and $\xi_{i}^{*}$ are slack variables that quantify the extent to which predictions fall outside the ε-insensitive tube, and C is the regularization parameter controlling the balance between model smoothness and error penalization.

To model nonlinear relationships in the microfluidics dataset, the Radial Basis Function (RBF) kernel was utilized, defined as:

(2) $$K\left(x,x^{\prime }\right)=exp(-\lambda {\left\|x-x^{\prime }\right\|}^{2})$$

where γ determines the locality or influence radius of each training sample in the feature space.

#### 2.2.2. Accuracy Assessment of ML Models

To quantitatively evaluate the predictive performance of the ML models, two standard statistical metrics were used: the coefficient of determination (R^2^) and root mean squared error (RMSE). These metrics characterize how closely the model predictions match the experimental measurements and whether the trained model generalizes well to unseen data.

The R^2^ measures the proportion of variance in the experimental data that is explained by the model and is defined as [44,45]:

(3) $$R^{2}=1-\frac{\sum_{i=1}^{n}{\left({ŷ}_{i}-y_{i}\right)}^{2}}{\sum_{i=1}^{n}{\left({ŷ}_{i}-y_{m}\right)}^{2}}$$

The RMSE, defined as the square root of the mean squared error, provides an error metric in the same units as the target variable:

(4) $$\mathrm{RMSE}=\sqrt{\frac{1}{n}\sum_{i=1}^{n}{\left({ŷ}_{i}-y_{i}\right)}^{2}}$$

where ${ŷ}_{i}$ and $y_{i}$ denote the predicted and actual values, respectively, for cumulative mass, the term $y_{m}$ represents average of the actual values, and n is the number of data samples.

RMSE is often preferred in regression model evaluation because it expresses error in the same physical units as the target variable, facilitating more intuitive comparison across models. Lower values of RMSE generally reflect improved predictive accuracy, while higher values of the R^2^ indicate that a larger proportion of variance in the experimental data is captured by the model. R^2^ is commonly used to assess how well the predictors explain the variability in the response; however, because R^2^ increases monotonically with the addition of input variables, it may overestimate model quality when redundant predictors are included.

When comparing overall regression performance across different model architectures, RMSE is typically more informative than R^2^, as it directly quantifies prediction error rather than the fraction of explained variance and thus offers a more reliable basis for model-to-model comparison [45,46,47]. Overall, lower RMSE values indicate improved predictive accuracy of the regression model, whereas higher R^2^ values (close to 1) reflect stronger explanatory power and are therefore preferred. Together, these metrics offer a comprehensive assessment of model accuracy and reliability across the microfluidic design library.

To further assess the robustness of the reported model-performance metrics, the configuration-level metric values were used to calculate the mean and standard deviation (SD) as follows [48]:

(5) $$\bar{x}=\frac{1}{n}\sum_{i=1}^{n}y_{i}$$

(6) $$SD=\sqrt{\frac{1}{n-1}\sum_{i=1}^{n}(y_{i}-\bar{y}{)}^{2}}$$

where $y_{i}$ is the value of the performance metric for configuration i, $\bar{y}$ is the mean value of the metric, and n=12 is the number of membrane—device configurations.

In addition, non-parametric bootstrap confidence intervals (CIs) were calculated to estimate the uncertainty of the mean model performance [49]. For each model and each metric, the twelve configuration-level values were randomly resampled with replacement 10,000 times. The mean value was recalculated for each bootstrap sample, generating a bootstrap distribution of the mean:

(7) $${\bar{y}}_{b}^{*}=\frac{1}{n}\sum_{i=1}^{n}y_{i,b}^{*}$$

where ${\bar{y}}_{b}^{*}$ is the mean of bootstrap sample b, $y_{i,b}^{*}$ is a resampled metric value, and b=1,2,…,10,000.

The 95% bootstrap confidence interval was then obtained from the 2.5th and 97.5th percentiles of the bootstrap distribution:

(8) $$CI_{95\%}=\left[P_{2.5}({\bar{y}}^{*}),P_{97.5}({\bar{y}}^{*})\right]$$

The final model-performance uncertainty was reported as mean ± standard deviation together with the 95% bootstrap CI. This analysis was used to evaluate whether the reported R^2^ and RMSE values were consistent across the twelve configurations rather than being driven by a single favorable case.

#### 2.2.3. SVR-Based Steady-State Identification and Operating-Point Selection

After training the SVR models with augmented data for each microfluidic design, a surrogate-based operating-point selection procedure was applied to identify the flow rate at which the system first reached steady-state cumulative mass. This procedure was data-driven and relied on SVR-predicted cumulative mass-time profiles rather than a formal numerical optimization algorithm.

In this work, “steady state” refers to an operational plateau in the SVR-predicted cumulative-mass profile. For each predicted cumulative-mass curve, a sliding-window linear regression was applied using a 20 min window. A curve was classified as reaching steady state when the absolute local slope remained below a relative tolerance of 0.02 × ΔC/20 min for a continuous duration of at least 15 min, where ΔC is the predicted cumulative-mass range of that curve. To avoid classifying trivial near-zero plateaus as steady state, the detected plateau also had to exceed 2% of the maximum predicted cumulative mass. When more than one flow rate satisfied these criteria, the flow reaching steady state earliest was selected, if two flows reached steady state at the same time, the higher cumulative-mass value was chosen.

**Figure 4:**
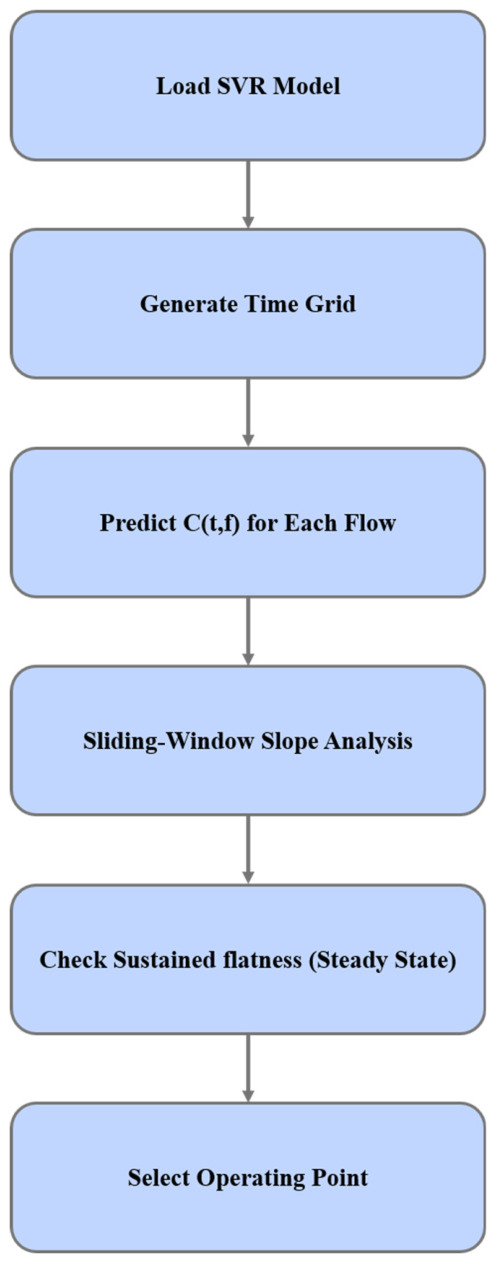
Workflow for SVR-based steady-state identification and operating-point selection.

This procedure was entirely data-driven, relying on SVR-predicted cumulative mass-time profiles rather than analytical or mechanistic models. Figure 4 illustrates the SVR-based steady-state identification and operating-point selection workflow.

For each design, the SVR model was loaded and evaluated on a dense, uniformly spaced time grid:

(9) $$t_{grid}=\left\{t_{min}-{∆t}_{pre},\ldots ,t_{max}+{∆t}_{post}\right\}$$

where $\Delta t_{\mathrm{pre}}$ and $\Delta t_{\mathrm{post}}$ allow optional extrapolation before and after the experimental range, respectively, and the grid spacing Δt is defined 1 min. For each candidate flow rate f, the SVR surrogate predicts the cumulative mass.

(10) $$\hat{C}(t,f)=\mathrm{SVR}(t,f)$$

Steady state is defined as the period in which the cumulative-mass curve becomes sufficiently flat. To quantify this, a linear regression slope is computed in sliding windows of length w minutes:

(11) $$\mathrm{slope}(k)=\frac{\sum_{i=1}^{n}\left(t_{i}-\bar{t}\right)(C_{i}-\bar{C})}{\sum_{i=1}^{n}{\left(t_{i}-\bar{t}\right)}^{2}}$$

where $t_{i}$ and $C_{i}$ are the points within the kth window, n is the number of samples in the sliding window, and w=win_len_min (20 min).

The tolerance for flatness is defined relative to the dynamic range of the signal:

(12) $$|\mathrm{slope}(k)|\leq \tau ,\tau =\alpha \;\frac{C_{max}-C_{min}}{w}$$

where α is a relative threshold which was set to 0.02. This design ensures that the definition of steady state scales naturally with the magnitude of the response.

The curve is considered to reach steady state only if the tolerance condition (Equation (8)) is satisfied for a continuous duration of at least h=15 min.

This prevents transient local fluctuations from being misclassified as steady state.

If a flat region is detected, the steady-state time and steady-state cumulative mass are defined as:

(13) $$t_{\mathrm{ss}}=\mathrm{first}\;\mathrm{time}\;\mathrm{at}\;\mathrm{which}\;\mathrm{the}\;\mathrm{flatness}\;\mathrm{is}\;\mathrm{sustained},C_{\mathrm{ss}}=\hat{C}(t_{\mathrm{ss}},f)$$

Additionally, the steady-state level must exceed a minimal fraction of the total concentration range:

(14) $$C_{\mathrm{ss}}\geq \beta \;(C_{max})(\beta =0.02)$$

This ensures that trivial plateaus near zero are ignored.

For each design, the algorithm evaluates all candidate flow rates ($f\in \left\{f_{1},f_{2},\ldots ,f_{m}\right\}$). A flow rate is considered feasible if a valid $\left(t_{\mathrm{ss}},C_{\mathrm{ss}}\right)$ pair exists. Among the candidate flow rates satisfying the steady-state criterion ($f^{*}$), the operating point was selected according to the following rule:

1. Earliest steady-state time

(15) $$f^{*}=arg\;\underset{f}{min}\;t_{\mathrm{ss}}(f)$$

2. Tie-breaker: higher steady-state cumulative mass

(16) $$\mathrm{maximize}\;C_{\mathrm{ss}}(f)$$

Thus, the selected flow rate provides a favorable balance between rapid steady-state attainment and high cumulative mass.

#### 2.2.4. Predictive Equations

Developing reliable models that accurately capture the relationships between key operating variables and process outputs is fundamental for analyzing, optimizing, and scaling chemical systems [23,50]. In this study, compact analytical surrogate models were generated for each membrane—microfluidic design to enable rapid prediction, steady-state operating-point selection, and integration into system-level simulations. These surrogate equations were constructed directly from the high-fidelity SVR models.

For every design, the previously trained SVR model with augmented data was reloaded and used to generate smooth, noise-free predictions across the experimental input domain. These SVR outputs served as supervisory targets for constructing explicit polynomial surrogate models. A symbolic-regression procedure based on a third-degree multivariate polynomial form was then applied to derive fully analytical correlations. The resulting polynomial surrogates retain the predictive fidelity of the SVR models while offering closed-form expressions that are computationally inexpensive, differentiable, and suitable for real-time optimization, sensitivity studies, and embedding into higher-level process simulators. This approach enables rapid exploration of operating and design conditions across the entire microfluidic architecture library.

If t represents the operation time and f is the flow rate, a multivariate polynomial expansion of degree d=3 was constructed as:

(17) $$\mathrm{cumulative}\;\mathrm{mass}=\Phi_{d}(t,f)$$

where $\Phi_{d}$ represents the complete polynomial basis up to degree three, including all cross-interaction terms:

(18) $$\Phi_{d}=\left\{t,f,t^{2},tf,f^{2},t^{3},t^{2}f,tf^{2},f^{3}\right\}$$

The resulting surrogate model takes the general form [51]:

(19) $$c\left(t,f\right)=\beta_{0}+\sum_{d=1}^{D}\beta_{d}\Phi_{d}(t,f)$$

where $\beta_{d}$ represents the coefficients estimated using ordinary least-squares or Ridge regression when regularization is required.

## 3. Results and Discussion

### 3.1. Machine Learning Model Comparison and Validation

For model development, the experimental datapoints were digitally extracted from [17] for three flow rates, providing cumulative-mass profiles over 5 h at 30 min intervals.

Table 3 presents the performance of six ML models across all microfluidic and membrane designs using CV and independent test metrics. Since each design contains only 33 experimental datapoints, dataset size strongly influences model behavior and interpretation. The CV scores indicate predictive ability on unseen subsets of the training data, while the independent test metrics, calculated on an untouched 20% hold-out set, provide the strictest indicator of held-out performance.

Across all configurations, SVR clearly outperformed the other algorithms, achieving the highest CV R^2^ values, typically 0.93–0.99, and markedly lower CV RMSE than tree-based models and KNN. More importantly, SVR maintained strong agreement between CV and test metrics, with test R^2^ values generally exceeding 0.97 and test RMSE often below 1–3 µg/cm^2^. For example, in PET–sMDC, SVR achieved a CV RMSE of 4.30 µg/cm^2^, improving to 0.78 µg/cm^2^ on the test set. Similarly, in CA–mMDC, SVR yielded a test R^2^ of 0.995 and a test RMSE of 0.91 µg/cm^2^, showing strong reconstruction of the observed transport profile even in designs with a low signal span.

By contrast, the other models showed consistent overfitting or sensitivity to data sparsity. Tree-based models such as XGB, GBR, and RFR often achieved moderate CV performance but degraded on the test set. For example, in PET-sMDC, XGB improves to a test R^2^ of 0.97 but retains a relatively high test RMSE of µg/cm^2^, while RFR performs poorly with a test RMSE of 14.6 µg/cm^2^. KNN also suffers from the sparse distribution of the datapoints, resulting in inconsistent test performance and inflated RMSE values across devices. The MLP consistently performs the worst, with negative CV R^2^ values and extremely large errors (e.g., RMSE values >60 µg/cm^2^ for PET-sMDC), reflecting the inability of neural networks to converge or generalize from such limited data.

**Figure 5:**
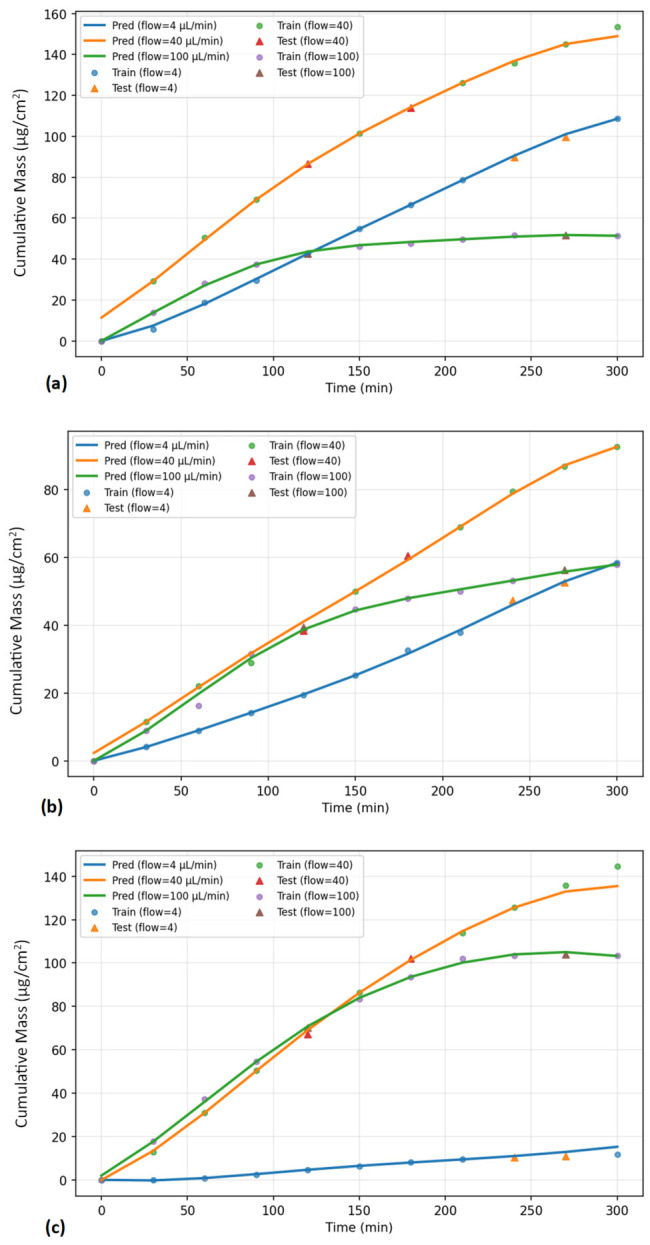
Caffeine cream permeation through a PET membrane at flow rates of 4, 40, and 100 µL/min in sMDC (**a**), mMDC (**b**), and LiveBox2 (**c**) devices.

**Figure 6:**
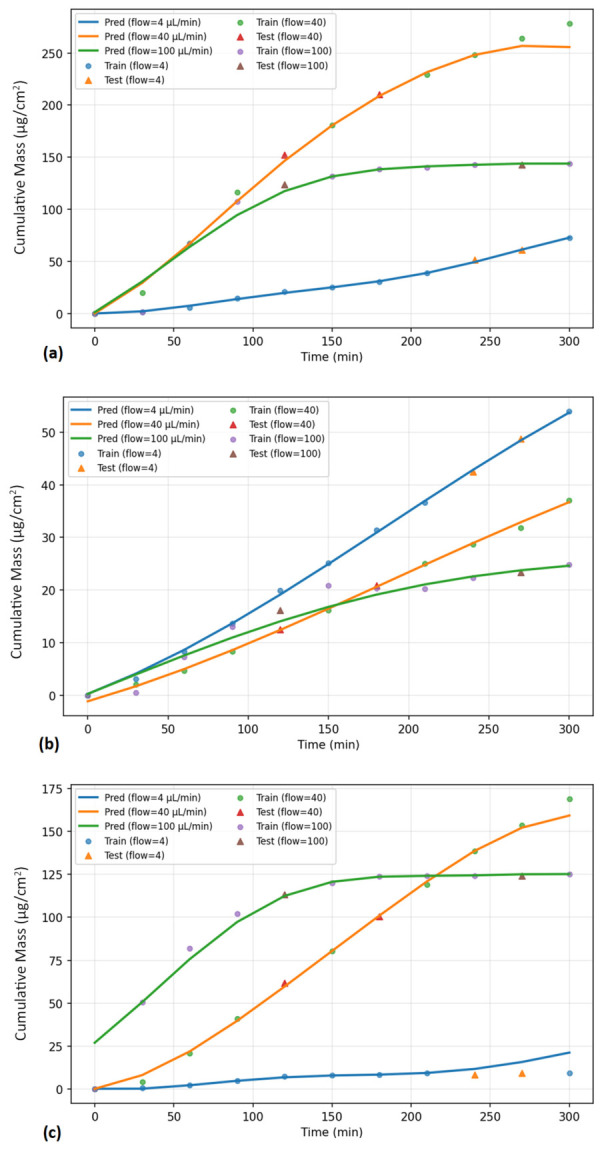
Caffeine cream permeation through a CA membrane at flow rates of 4, 40, and 100 µL/min in sMDC (**a**), mMDC (**b**), and LiveBox2 (**c**) devices.

**Figure 7:**
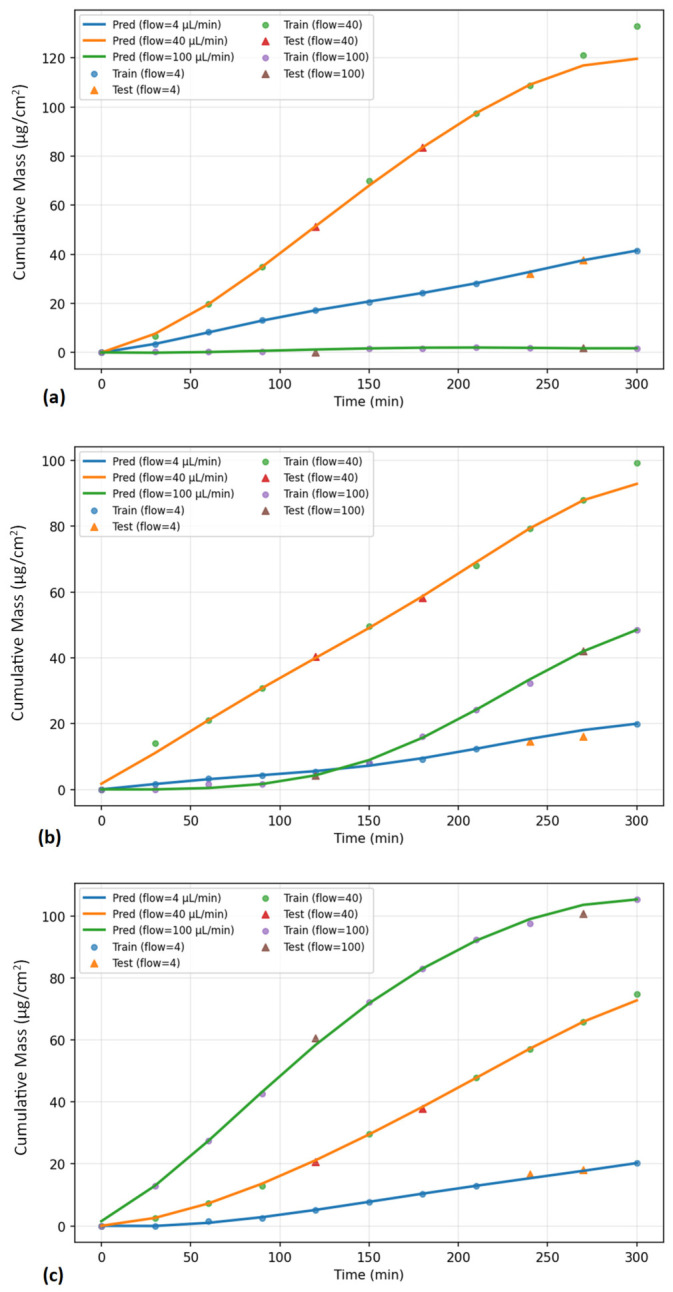
Caffeine cream permeation through a rat skin membrane at flow rates of 4, 40, and 100 µL/min in sMDC (**a**), mMDC (**b**), and LiveBox2 (**c**) devices.

**Figure 8:**
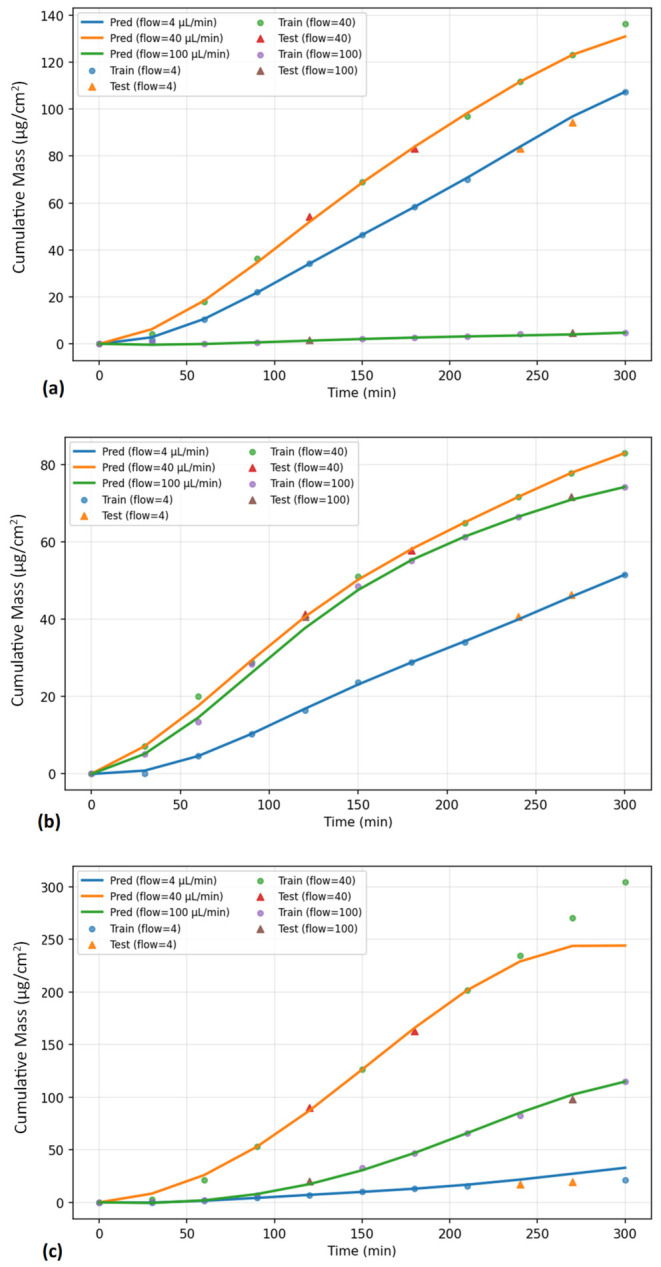
Caffeine cream permeation through an alginate membrane at flow rates of 4, 40, and 100 µL/min in sMDC (**a**), mMDC (**b**), and LiveBox2 (**c**) devices.

Overall, Table 3 demonstrates that SVR was the most effective model for reproducing the smooth, monotonic cumulative-mass evolution characteristic of TTDS diffusion profiles with only 33 datapoints per system. The strong agreement between CV and test metrics indicates that SVR provides a suitable balance between flexibility and regularization, whereas higher-capacity models (MLP, XGB, GBR, and RFR) could not maintain stable generalization in this data-scarce regime. Therefore, SVR was selected for detailed validation against the experimental TTDS datasets reported in [17]. Figure 5, Figure 6, Figure 7 and Figure 8 show the SVR-predicted cumulative mass of caffeine cream at flow rates of 4, 40, and 100 µL/min alongside the experimental measurements. In all microfluidic configurations—(a) sMDC, (b) mMDC, and (c) LiveBox2—the solid lines show SVR predictions while the symbols represent the experimental training points (filled circles) and the held-out test points (triangles). Across all membranes and microfluidic devices, as indicated in figures, the SVR predictions follow the experimental trends with high fidelity, capturing the correct curvature, magnitude, and separation between the three flow-rate profiles.

The unseen test points fall directly on the predicted curves, demonstrating that the model learned a smooth, physically consistent time-flow-permeation mapping rather than memorizing individual datapoints. This behavior is fully consistent with the high CV and test R^2^ values obtained for each design, despite the limited dataset of only 33 datapoints per configuration.

For the PET membrane (see Figure 5), the SVR model reproduces the caffeine transport behavior reported experimentally. At 4 µL/min, slow medium renewal causes rapid receptor-phase accumulation and decay of the concentration gradient, resulting in the lowest cumulative-mass curves. Increasing the flow to 40 µL/min enhances convective refreshment and maintains a strong driving force, giving rise to the highest permeation rate. As reported in the original study, the cumulative mass at 40 µL/min becomes 1.7–12.9 times higher than at 4 µL/min across the three devices, and the SVR predictions reproduce this with high accuracy. At 100 µL/min, the residence time becomes insufficient for complete diffusive uptake, causing the cumulative mass to fall below the 40 µL/min flow rate, another trend recovered accurately by the SVR curves. For CA (Figure 6), the SVR model again reproduces the experimentally observed flow-rate hierarchy (40 > 100 > 4 µL/min), the curvature of the permeation profiles, and the device-specific differences arising from the three microfluidic geometries. In the sMDC configuration (Figure 6a), which has the smallest effective diffusion area and internal volume, the cumulative-mass profiles show the largest separation between the flow rates, with 40 µL/min exceeding 250 µg/cm by the end of the experiment. The SVR model captures this pronounced flow sensitivity with excellent accuracy. In mMDC (Figure 6b), the membrane area is larger and the receptor volume smaller, which reduces the transmembrane resistance and leads to overall higher fluxes; however, the enhanced diffusion area also diminishes the relative differences between the three flow rates because the system becomes less limited by receptor-phase accumulation. Consequently, the experimental curves lie closer together, and the SVR model correctly reproduces this reduced flow-rate dependence without introducing artificial oscillations. LiveBox2 (Figure 6c) exhibits intermediate behavior: its larger chamber volume delays saturation but still maintains clear enhancement at 40 µL/min. Here too, the SVR prediction accurately follows the strong early-time rise and the gradual late-time flattening of the 40 µL min^−1^ profile.

Rat skin (see Figure 7) exhibits the lowest permeability among all tested membranes, and the SVR model correctly reflects the slow and strongly diffusion-limited cumulative-mass trends characteristic of biological tissue. The curves show long lag times, shallow slopes, and low final cumulative masses, all of which are predicted accurately by the model. At 40 µL/min, permeation becomes moderately enhanced, and the SVR curves track this increase. The effect of 100 µL/min is markedly device-dependent: in sMDC (Figure 7a) and mMDC (Figure 7b), the limited residence time reduces permeation compared to that at 40 µL/min, while in LiveBox2 (Figure 7c) the larger receptor volume compensates for the reduced contact time and produces the highest cumulative mass. The SVR predictions capture this cross-over behavior with exceptional precision, consistent with the extremely high test R^2^ values (0.997–1.000) obtained for rat skin datasets.

The alginate scaffold membrane (Figure 8) displays the highest permeability due to its hydrated polymer network. The SVR predictions reproduce the steep slopes at intermediate times and the high final cumulative masses observed experimentally. At 4 µL/min, permeation increases steadily, while at 40 µL/min the refreshment rate is optimal, producing the largest cumulative mass in sMDC (Figure 8a) and mMDC (Figure 8b). In LiveBox2 (Figure 8c), however, the 100 µL/min condition becomes the highest, compared to other devices, due to the larger membrane area and receptor volume that mitigate residence-time losses. The SVR curves mirror these device-specific behaviors accurately, including the early rapid rise and late-time saturation characteristic of alginate systems. The close alignment with both training and test points highlights the model’s ability to reproduce the underlying physicochemical behavior of highly permeable hydrogel membranes.

The uncertainty analysis, as represented in Table 4, further confirms that SVR provides the most accurate and stable performance across the twelve membrane—device configurations. SVR achieved the highest mean test R^2^ of 0.994 ± 0.0079, with a 95% bootstrap CI of 0.989–0.997. It also produced the lowest mean test RMSE of 1.800 ± 1.258, with a 95% bootstrap CI of 1.180–2.537. The small SD and narrow CI of the SVR test R^2^ indicate that the high predictive performance was consistent across the tested configurations rather than being driven by a single favorable case. In comparison, MLP and RFR showed substantially larger RMSE values and wider variability, indicating lower reliability for the present small dataset. These results support the selection of SVR as the most stable in-domain surrogate model for the investigated caffeine permeation profiles.

Overall, Table 3 and Table 4, and Figure 5, Figure 6, Figure 7 and Figure 8 show that SVR provides strong predictive capability across the four membrane types and three device geometries, accurately reproducing the observed effects of flow rate, residence time, membrane permeability, and cumulative mass despite the limited dataset of 33 datapoints per design. These results support SVR as a robust surrogate model for TTDS transport behavior in microfluidic membrane-diffusion systems.

Although the SVR models reproduced the cumulative-mass profiles with high fidelity, minor systematic deviations were observed in a few cases. These mainly occurred in low-permeability systems such as rat skin, where long lag times led to slight early-time underestimation, and in highly permeable systems such as alginate-LiveBox2, where sharp curvature caused mild over-smoothing near inflection regions. These deviations did not affect the overall predictive trends, but they indicate that SVR may be less accurate when abrupt transport changes are sparsely represented in the training data. No major model failures were observed, and Gaussian-noise augmentation helped reduce sensitivity to isolated datapoints.

### 3.2. Performance Evaluation of the SVR Model

Table 5 summarizes the SVR performance after applying Gaussian-noise data augmentation and compares it with the baseline results obtained from the original, non-augmented dataset. Furthermore, the final optimized SVR hyperparameters for each of the twelve configurations are presented in Table S2. Because each membrane-device configuration contains only 33 experimental measurements, even small perturbations in the data distribution can influence CV behavior.

Augmentation therefore provides a controlled means of enriching the sampling density and evaluating the stability of the SVR model under slightly perturbed but experimentally bounded training conditions.

The most noticeable effect of augmentation appears in the cross-validation metrics. In nearly all configurations, the CV R^2^ values increase and converge toward very high levels (0.992–0.998), indicating that the augmented dataset enables the model to learn a smoother and more internally coherent representation of the cumulative-mass dynamics. The corresponding CV RMSE values, however, do not uniformly decrease: in some cases, they are reduced (e.g., PET-sMDC: 4.31 → 2.25 µg/cm^2^), while in others they increase (e.g., PET-mMDC: 2.39 → 6.95 µg/cm^2^). This behavior is expected because augmentation locally densifies the data around each measurement point, and depending on the curvature of the underlying cumulative-mass profile, this can either reinforce or slightly distort the local neighborhood structure. As such, CV metrics primarily reflect the model’s response to a denser, but also noisier, representation of the input manifold rather than a uniform increase in intrinsic predictive accuracy.

Despite this mixed CV behavior, augmentation provides a clear benefit in stabilizing the SVR model and preventing the large error spikes observed in the original dataset. Without augmentation, several membrane–device combinations exhibit comparatively high RMSE values; for example, CA-sMDC reaches 14.8 µg/cm^2^, CA-LiveBox2 reaches 9.1 µg/cm^2^, and alginate-LiveBox2 reaches 18.9 µg/cm^2^. These elevated errors stem from the limited number of datapoints sampled at only three flow rates, which leaves broad regions of the input–output space sparsely populated and forces the model to rely heavily on a small number of influential points.

After augmentation, these error spikes disappear entirely. Across all membranes and devices, the RMSE values collapse into a narrow, physically reasonable range of approximately 1–4 µg/cm^2^, which lies well within the natural variability of the cumulative-mass profiles themselves (typically 50–300 µg/cm^2^ depending on membrane type and geometry). This improvement highlights the principal advantage of augmentation: by enriching each neighborhood of the dataset with small, physically plausible perturbations, the effective training distribution becomes both denser and smoother. Consequently, the SVR solution, determined by the set of support vectors, is no longer dominated by a few isolated datapoints but instead anchored to a broader and more representative approximation of the underlying cumulative-mass surface. This leads to more uniform CV and test behavior and markedly reduces the model’s sensitivity to data sparsity.

Importantly, augmentation does not artificially inflate accuracy or reduce the meaningful test error beyond physically plausible limits. Rather, it suppresses unstable, high-variance behavior and yields a more robust, well-regularized model whose performance remains consistently high across membranes and device geometries. This demonstrates that although SVR already provides excellent predictive accuracy for data-limited TTDSs, Gaussian-noise augmentation enhances model reliability and effectively eliminates the high-RMSE outliers observed in the non-augmented results.

In addition to Table 5, prediction-error plots for all membrane–device combinations are provided in Supplementary Information (Figures S1–S4). These plots show an excellent alignment between predicted and experimental cumulative-mass values, with best-fit regression lines lying almost exactly on the identity line and R^2^ values approaching unity. The tight clustering of points demonstrates that the SVR model captures the underlying transport behavior with high fidelity and minimal systematic bias, fully consistent with the low RMSE values reported in Table 5.

**Figure 9:**
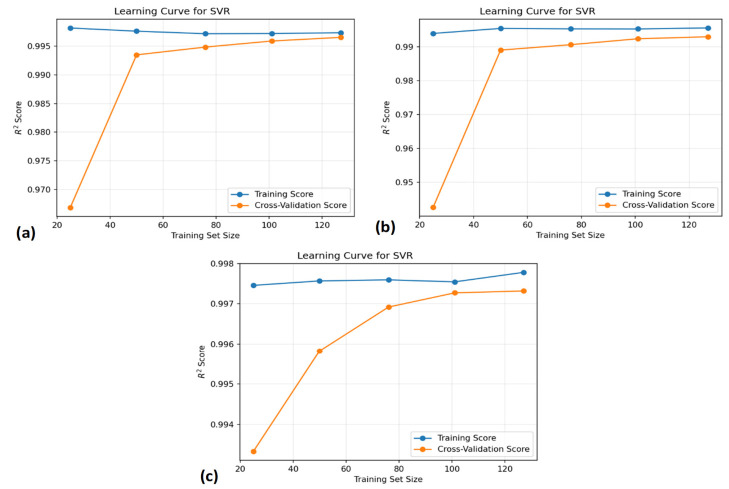
Learning curves for the PET membrane in sMDC (**a**), mMDC (**b**), and LiveBox2 (**c**) devices.

**Figure 10:**
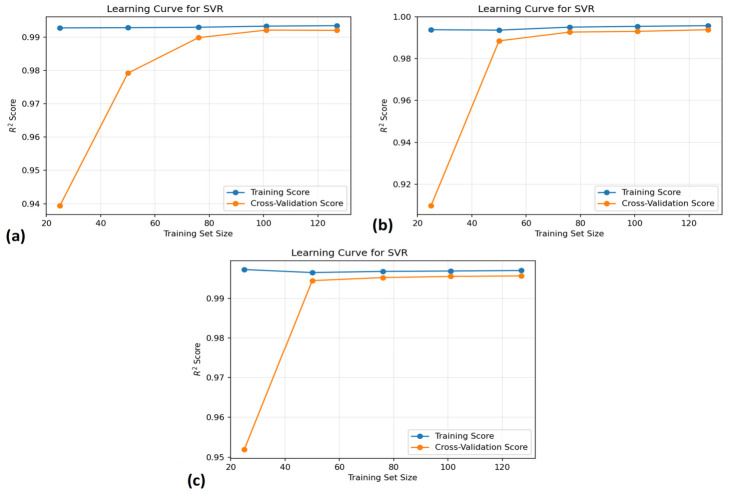
Learning curves for the CA membrane in sMDC (**a**), mMDC (**b**), and LiveBox2 (**c**) devices.

**Figure 11:**
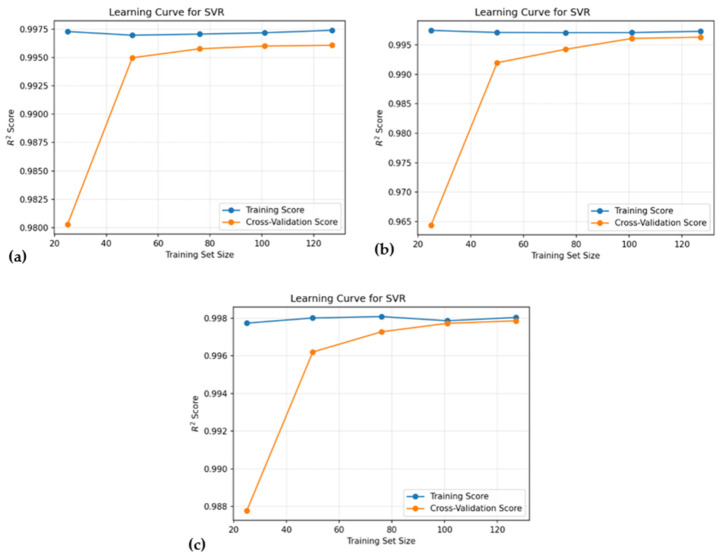
Learning curves for the skin rat membrane in sMDC (**a**), mMDC (**b**), and LiveBox2 (**c**) devices.

**Figure 12:**
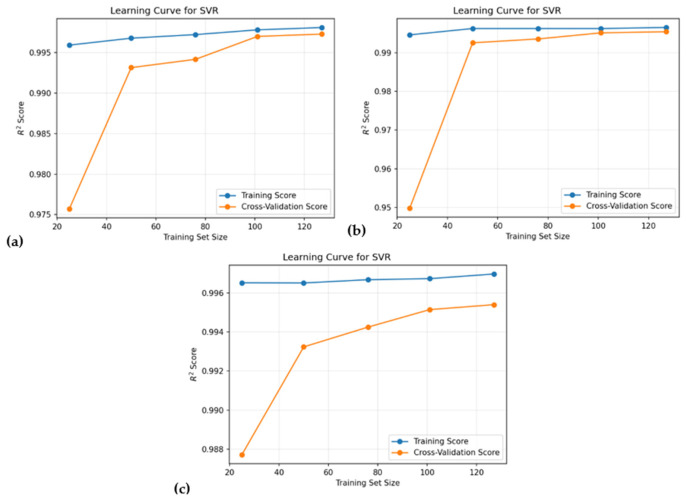
Learning curves for the alginate scaffold membrane in sMDC (**a**), mMDC (**b**), and LiveBox2 (**c**) devices.

Figure 9, Figure 10, Figure 11 and Figure 12 present the learning curves used to assess potential overfitting across the four membranes and three microfluidic devices. Figure 9 shows that for the PET membrane, the SVR model exhibits highly stable learning behavior with no indication of overfitting.

Training R^2^ scores remain consistently high (≈0.996–0.998), while the CV R^2^ increases smoothly as additional samples are included, ultimately converging toward the training performance. This behavior is characteristic of SVR trained on sparse experimental data, where adding more points helps define the underlying nonlinear mapping between time, flow rate, and cumulative mass. The larger initial gap between training and CV scores reflects the limited sample size which increases variance at small training fractions.

As the training set grows, this gap narrows and the curves flatten, demonstrating excellent generalization. The consistently high and nearly parallel curves across all devices confirm that PET diffusion that is characterized by smooth, monotonic cumulative-mass profiles is accurately captured by the SVR model.

Figure 10 shows similarly robust learning behavior for the CA membrane, although with slightly more pronounced initial gaps due to the stronger curvature and steeper transport dynamics of CA. When only ~20% of the dataset is used, CV R^2^ values begin at lower levels, but they rise rapidly with additional training samples and converge toward the training curve as the dataset becomes more complete. These trends demonstrate that SVR effectively reconstructs the nonlinear shape of the CA permeation trends once enough samples are available. The nearly flat training curves and the consistent convergence of CV scores (approaching 0.99–1.00) across all devices indicate that the SVR model remains well balanced and does not overfit, even when trained on Gaussian-augmented data. Overall, the curves confirm that the model yields a stable and physically meaningful surrogate for CA transport despite the limited experimental sampling.

The learning curves in Figure 11 indicate that rat skin permeation, despite its higher diffusional resistance and more complex biological structure, is also learned effectively by the SVR model. Training R^2^ scores remain very high (≈0.995–0.998), while CV R^2^ values, initially lower due to early lag phases and sharper gradients in the skin permeation profiles, increase steadily with training size and ultimately converge with the training performance. The slightly broader gaps at smaller training sizes are consistent with the greater complexity and nonlinearity of biological membranes compared to synthetic ones. Nevertheless, once ~60–80% of the training data are included, CV curves align closely with training curves, demonstrating that SVR captures the skin-transport dynamics accurately while remaining well regularized across devices.

Figure 12 shows that the SVR model also generalizes well for the alginate scaffold, the membrane with the steepest and most flow-sensitive diffusion profiles. The CV R^2^ starts at moderate values for small training sizes, reflecting the strong nonlinearity and large separation between flow-rate conditions, but increases rapidly and converges toward the training curve as more samples are added. Training R^2^ values remain consistently high, and no divergence or instability is observed, indicating that the margin-based structure of SVR prevents overfitting even under high curvature. The near-overlap between training and cross-validation curves at larger sample sizes confirms that the model successfully captures the sharp, high-permeability transport behavior of alginate membranes, offering a robust and physically consistent surrogate even under limited-data conditions. In addition to R^2^-based learning curves, training and validation RMSE learning curves were generated and are provided in Supplementary Information (Figures S5–S8) to further evaluate model convergence and potential overfitting.

### 3.3. Effect of Parameters on the Drug Delivery System

#### 3.3.1. Predictive Equations for Cumulative Mass

The cubic polynomial correlations in Table 6 provide explicit surrogate models for the SVR predictions, expressing the cumulative mass of caffeine as a function of time (t, min) and flow rate (f, µL/min) for each membrane–device configuration. Each equation contains a constant term, linear terms in t and f, all second-order contributions (t^2^, tf, f^2^), and the complete third-order interaction set (t^3^, t^2^f, tf^2^, f^3^). This structure allows the polynomials to reproduce the smooth but strongly nonlinear transport surfaces learned by the SVR models while remaining fully analytical. Furthermore, the R^2^ values quantifying the agreement between the cubic polynomial equations and the corresponding SVR predictions are presented in Table S3.

The first-order coefficients in t and f dominate the early-time behavior: positive t coefficients correspond to the initial linear growth of cumulative mass with exposure time, whereas positive f coefficients quantify the first-order enhancement of transport with increasing perfusion rate.

The second-order terms introduce curvature and encode the balance between diffusion, membrane resistance, and hydrodynamics. A negative t^2^ coefficient, as seen for several PET and CA cases, represents a gradual deceleration of cumulative uptake at long times, consistent with depletion of the driving concentration gradient across the membrane. Positive tf terms capture the synergistic effect of time and flow rate, where prolonged exposure at moderate-to-high perfusion sustains a stronger gradient and leads to higher cumulative mass. The f^2^ terms reflect how strongly the system responds to changes in flow rate: in highly permeable configurations such as alginate–LiveBox2, relatively large |f^2^| values indicate a pronounced, nonlinear dependence of permeation on perfusion. In more diffusion-limited systems, such as rat skin in the compact mMDC device, the f^2^ and tf coefficients are smaller in magnitude, indicating that increasing flow rate beyond a certain level yields diminishing returns because the skin barrier, rather than convective renewal, becomes rate-controlling.

The third-order terms provide fine adjustment of the surface shape and are essential for capturing subtle features of the SVR predictions, particularly the combination of early linear growth, mid-time acceleration or deceleration, and eventual approach to a quasi-plateau. Terms in t^3^ and t^2^f control the late-time bending of the curves, allowing the polynomial to mimic saturation behavior without imposing an explicit mechanistic model, while tf^2^ and f^3^ terms reproduce the experimentally observed non-monotonic dependence on flow (e.g., 40 µL/min outperforming 4 and 100 µL/min in several geometries). The systematic variation in coefficient magnitudes across all configurations is physically consistent: more permeable membranes (PET, alginate) and devices with a larger diffusion surface (LiveBox2) tend to have larger positive t, tf, and f^2^ contributions, reflecting higher fluxes and stronger flow sensitivity, whereas rat skin and the smaller chambers show weaker or partially compensating higher-order terms associated with lower permeability and stronger diffusion control.

Together, these cubic correlations act as compact, physics-consistent surrogates of the SVR models. They retain the predictive fidelity of the ML framework but can be evaluated almost instantaneously, easily differentiated with respect to t and f, and directly embedded into optimization, control, or system-level simulators. This makes them particularly attractive for rapid design-space exploration (e.g., identifying optimal flow rates and exposure times), sensitivity analysis, or coupling with larger pharmacokinetic or device-scale models, while preserving the experimentally validated transport behavior encoded in the original TTDS microfluidic data.

#### 3.3.2. Surrogate-Based Steady-State Operating-Point Selection

Table 7 summarizes the SVR-based identification of the selected flow rate, the time at which steady state is reached (t_ss_), and the corresponding steady-state cumulative mass (c_ss_) for each membrane and microfluidic device. These values were obtained using the automated steady-state detection procedure, which evaluates SVR-predicted permeation curves across a fine time grid and determines the earliest time interval where the predicted slope falls below a noise-adjusted tolerance.

For PET, the SVR-based operating-point selection consistently identified 100 µL/min as the selected flow rate in all three devices, reflecting PET’s low permeability and strong dependence on convective refreshment to maintain the concentration gradient. Although mMDC has a larger diffusion surface than sMDC, its geometry also includes a larger receptor region and a lower effective driving force per unit volume, which causes the cumulative-mass curve to rise more gradually. As a result, mMDC reaches steady state later (≈302 min) despite its larger membrane area. In contrast, sMDC, with a smaller receptor volume, saturates more quickly and therefore reaches steady state earlier (≈178 min). LiveBox2, despite having the largest membrane area, shows an intermediate steady-state time (≈223 min) because the larger volume delays concentration buildup while the higher area increases flux. The combined effect produces a smoother, more balanced approach to steady state. Thus, the differences in steady-state times across devices arise primarily from geometry- and volume-driven mass transfer dynamics, not from membrane area alone.

The CA membrane exhibits intermediate permeability, and its selected flow rate varies strongly with device geometry because convection and residence time influence CA transport differently across architectures. In sMDC, the smallest device with the lowest receptor volume, the selected flow rate was 4 µL/min, not because high flow reduces mass transfer, but because in this geometry the receptor volume saturates rapidly. At higher flow rates, the medium is refreshed so aggressively that the CA membrane, which is already reasonably permeable, cannot fully exploit the concentration gradient before the receptor fluid is replaced. As a result, the cumulative-mass curve becomes flatter and the selected operating point shifts to low flow.

In mMDC and LiveBox2, however, the situation reverses. These devices have larger receptor volumes and diffusion surface, meaning that concentration buildup is slower and the gradient can be maintained for longer. Under these conditions, higher flow rates (100 µL/min) do not prematurely erase the gradient; instead, they help sustain it by continually removing permeated solute. Consequently, CA reaches its highest steady-state mass at 100 µL/min in mMDC and LiveBox2. This geometry-dependent shift highlights that CA transport is governed by a delicate coupling between its moderate permeability and the device-specific hydrodynamic renewal rate.

Rat skin shows the highest diffusional resistance among all membranes, and its selected flow behavior is dominated by the need to maintain sufficient residence time for solute to cross the multilayered barrier. In sMDC and mMDC, where receptor volumes are relatively small and gradients decay more quickly, the selected flow rate appears at 40 µL/min. At the lower flow (4 µL/min), renewal is too slow and the gradient decays; at the higher flow (100 µL/min), the receptor fluid is replaced too rapidly, and the solute cannot penetrate the skin’s barrier quickly enough to be captured in the outlet. In LiveBox2, the picture changes. Its much larger membrane area and receptor volume slow down gradient decay and extend the effective contact time per unit volume. As a result, the higher flow (100 µL/min) becomes favorable because it enhances convective removal without excessively shortening residence time. This explains why LiveBox2 achieves its selected flow rate at 100 µL/min, while smaller devices do not. The notably long steady-state times (up to 420 min) reflect the inherently slow, diffusion-limited transport characteristic of biological tissue.

Alginate is the most permeable membrane evaluated, and its steady-state selection pattern reflects its highly hydrated, low-resistance structure. In sMDC and LiveBox2, where the gradient can be sustained over long durations, the selected flow rate emerges at 40 µL/min. Here, alginate’s high permeability means that moderate flow provides a favorable balance: the solute diffuses readily, and the receptor fluid is renewed quickly enough to maintain the gradient but not so fast that residence time is sacrificed. In mMDC, however, the larger diffusion area relative to volume shifts the selected flow rate to 100 µL/min, because the device geometry allows strong convective refreshment to maintain high gradients over a larger membrane surface. Thus, the selected flow rate depends on how device geometry interacts with the extremely high permeability of the hydrogel. Notably, alginate in LiveBox2 produces the highest steady-state cumulative mass in the entire dataset (≈448 µg/cm^2^), illustrating the synergistic effect of a high-permeability membrane combined with a large-area, large-volume device.

Taken together, the SVR-based steady-state operating-point analysis indicates that the alginate scaffold is the most suitable membrane for TTDSs among the materials investigated, owing to its consistently superior permeability and robust performance across microfluidic device geometries. In every configuration, alginate achieves substantially higher steady-state cumulative masses than PET, CA, or rat skin, reaching values as high as ≈448 µg/cm^2^ in LiveBox2, which is the highest of the entire dataset. This reflects alginate’s highly hydrated, low-resistance hydrogel structure, which supports rapid solute diffusion and maintains strong concentration gradients even under moderate flow conditions (40–100 µL/min). Unlike CA and PET, which exhibited more pronounced device-dependent operating points because of their moderate permeability, and unlike rat skin, which suffers from long lag times and limited flux, alginate maintains high transport rates without requiring fine-tuning of flow or geometry, indicating a wider operational robustness for drug delivery applications. Furthermore, the ability of alginate to achieve high c_ss_ values across devices, particularly in large-volume configurations such as LiveBox2, highlights its potential for both screening studies and scalable TTDS platforms. Therefore, based on the combined modeling and steady-state operating-point analysis, alginate provides the most favorable balance of permeability, operational flexibility, and cumulative drug release, making it the most promising membrane for efficient TTDS performance in microfluidic diffusion systems.

### 3.4. Limitations and Assumptions of the Proposed Machine Learning Framework

The proposed ML framework was developed using permeation data obtained under a limited set of operating conditions, specifically three PPF flow rates and a defined experimental time window. Consequently, the model is primarily intended to describe and interpolate permeation behavior within this experimentally studied domain. This assumption is consistent with the observed data structure, where cumulative permeation curves exhibited smooth and monotonic trends that are well suited for regression-based learning approaches. Although extrapolation beyond the trained flow rate range or time window should be interpreted with caution, the model demonstrates strong performance within the studied conditions. Accordingly, the present models should be viewed as configuration-specific in-domain surrogate models for interpolation and smoothing within the experimentally investigated caffeine-membrane-device-flow domain, rather than as universal predictors for unseen drugs, membranes, devices, or biological systems. In addition, because caffeine is a small and relatively hydrophilic molecule, the quantitative predictions reported here should be considered most applicable to caffeine or caffeine-like compounds. Extension of this workflow to other transdermal delivery systems would require new experimental data, model retraining, and independent validation, ideally incorporating additional descriptors such as membrane thickness, porosity, diffusion area, active-compound molecular weight, logP, pKa, solubility, ionization state, diffusivity, and formulation composition. Thus, the proposed framework is repeatable and adaptable, but its current trained models are specific to the tested caffeine permeation dataset. In particular, the SVR approach was selected because of its robustness in small-data regimes and its ability to generate smooth empirical interpolations within the experimentally studied domain. This makes it well suited for reconstructing nonlinear permeation profiles when experimental sampling is sparse.

Another consideration is that the ML model does not explicitly incorporate the governing transport equations. However, the learned relationships remain physically plausible because the model was trained directly on experimentally measured permeation profiles and constrained through bounded data augmentation that preserved the original curve structure. As a result, the framework provides a reliable data-driven approximation of the experimentally observed behavior while avoiding overfitting.

Finally, while the dataset size is inherently limited due to the experimental complexity of microfluidic diffusion studies, the combination of SVR modeling and controlled Gaussian-noise augmentation enabled stable learning and accurate reconstruction of permeation dynamics.

## 4. Conclusions and Future Trends

This study presents a data-driven ML framework for modeling, steady-state identification, and operating-point selection in microfluidic transdermal drug-delivery systems, integrating four membrane types (PET, CA, rat skin, and alginate scaffold) and three microfluidic devices (sMDC, mMDC, and LiveBox2). Using a limited set of only 33 experimental datapoints per configuration, six ML models, including MLP, GBR, XGB, KNN, RFR, and SVR, were systematically evaluated to determine their predictive capability. Among these, SVR consistently demonstrated the highest accuracy (test R^2^ > 0.97 and RMSE < 1–3 µg/cm^2^), enabling precise reconstruction of cumulative-mass curves across all flow rates (4, 40, 100 µL/min) within each experimental membrane–device configuration.

The SVR models effectively captured the nonlinear effects of flow rate and membrane permeability on cumulative mass. The alginate membrane stood out, showing the highest steady-state cumulative mass across all devices. It reached 447.96 µg/cm^2^ in the LiveBox2 system and 142.91–305.50 µg/cm^2^ in sMDC/mMDC, confirming its superior permeability and suitability for TDD applications. PET and CA membranes had lower permeation, which is consistent with their polymeric barrier properties. The type of device also played a significant role in mass transport. Typically, mMDC achieved faster renewal and higher cumulative mass than sMDC.

Using the trained SVR surrogates, favorable operating conditions were selected through an automated steady-state detection and operating-point selection procedure. Across several configurations, 100 µL/min proved to be the most effective flow rate for achieving steady state rapidly while maximizing cumulative mass. For example, PET membranes in the mMDC system reached a steady-state cumulative mass of 57.63 µg/cm^2^, while CA membranes in LiveBox2 achieved 121.16 µg/cm^2^ at steady state. These predictions provide valuable guidance for selecting microfluidic operating ranges in future TDD studies.

Additionally, concise third-degree polynomial surrogate equations were developed for each device—membrane setup. These equations provide analytical expressions that closely match SVR predictions. They allow for rapid simulation, optimization, and integration into larger in silico pharmacokinetic or device-design workflows.

Overall, this work shows that ML-driven modeling, especially SVR combined with Gaussian-noise augmentation, can improve in-domain predictive accuracy, reduce experimental burden, and enable automated steady-state identification and data-driven operating-condition selection within experimentally characterized microfluidic TDD systems.

The findings of this study open several promising directions for advancing ML-assisted microfluidic evaluation of TDDSs. A natural next step is extending the current framework beyond a single drug molecule. This could involve including a diverse range of therapeutic compounds with different physicochemical properties. By adding parameters like molecular weight, solubility, formulation composition, and excipient interactions, more generalized predictive models that can handle multicomponent and clinically relevant formulations can be developed. Another key area is merging the surrogate equations with mechanistic transport models. Hybrid models that link data-driven learning with Fickian diffusion, multilayer skin models, or CFD-based hydrodynamic simulations would enhance predictive capabilities. This would allow researchers to explore untested geometries, membrane designs, and operating conditions without needing more experiments. Such models could also facilitate physiologically relevant predictions by accounting for changes in skin hydration, thickness, age, or medical conditions, paving the way for personalized TDD optimization. Lastly, the workflow developed here is suitable for automation. Combining microfluidic experiments with ML-guided active learning strategies could turn membrane screening and formulation optimization into a high-throughput, adaptive process. At the same time, integrating the polynomial surrogate models into microfluidic controllers could allow for real-time adjustments of flow profiles or drug-release conditions, creating smart TDD platforms with closed-loop control.

## Figures and Tables

**Table 1:** Technical characteristics of the microfluidic diffusion systems and diffusion platforms [17].

Device Type	Diffusion Surface Area (cm^2^)	Compatible Membranes	Membrane Platform Thickness (mm)
sMDC mMDC LiveBox 2	0.283 0.503 1.767	PET membrane CA membrane Rat skin Alginate hydrogel scaffold	0.012 0.20 0.59 1.77

**Table 2:** Controlled, measured, and derived parameters in TDDS–microfluidics experiments.

Category	Parameter	Description
Controlled parameters	Device geometry	sMDC, mMDC, and LiveBox2
	Membrane type and thickness	PET, CA, rat skin, and alginate; thickness values shown in Table 1
	PPF flow rate	4, 40, and 100 μL/min
	Donor formulation	Caffeine cream formulation applied to the donor channel
	Channel layout and membrane contact area	Fixed per device design; determining the mass-transfer area
Measured quantities	Cumulative mass	Caffeine mass collected at the receiver outlet
	Time-series sampling	Measurements recorded at fixed time intervals throughout 300 min
Derived quantities	Diffusion profiles	Cumulative mass vs. time curves for each configuration
	Transport kinetics	Flow- and membrane-dependent permeation behavior computed from experimental curves

**Table 3:** Cross-validation and training of regression models for various microfluidic devices and membrane configurations.

Membrane	Device	Model	CV R^2^	CV RMSE	Test R^2^	Test RMSE
PET	sMDC	SVR	0.987	4.305	0.999	0.784
		XGB	0.860	12.937	0.970	4.436
		GBR	0.931	9.438	0.961	5.008
		KNN	0.843	12.459	0.951	5.625
		RFR	0.726	17.413	0.671	14.589
		MLP	0.001	25.263	−5.113	62.876
	mMDC	SVR	0.981	2.394	0.972	1.373
		XGB	0.890	6.668	0.813	3.557
		GBR	0.821	7.628	0.747	4.136
		KNN	0.908	4.998	0.677	4.669
		RFR	0.881	7.153	0.397	6.380
		MLP	0.814	9.385	−8.027	24.686
	LiveBox2	SVR	0.985	4.790	0.999	1.309
		XGB	0.912	12.539	0.988	4.232
		GBR	0.964	7.667	0.986	4.504
		KNN	0.979	6.882	0.967	6.937
		RFR	0.908	13.620	0.783	17.746
		MLP	0.689	24.023	0.087	36.398
CA	sMDC	SVR	0.959	14.786	0.996	3.537
		XGB	0.852	25.232	0.961	10.748
		GBR	0.879	22.989	0.940	13.352
		KNN	0.966	14.925	0.884	18.546
		RFR	0.856	28.974	0.630	33.109
		MLP	0.275	63.631	−0.387	64.074
	mMDC	SVR	0.963	1.888	0.995	0.913
		XGB	0.872	4.011	0.976	2.066
		GBR	0.785	5.172	0.975	2.115
		KNN	0.859	4.198	0.913	3.973
		RFR	0.790	5.066	0.905	4.157
		MLP	0.789	3.624	−1.303	20.442
	LiveBox2	SVR	0.963	9.066	0.996	3.113
		XGB	0.919	15.471	0.989	5.044
		GBR	0.898	16.813	0.985	5.808
		KNN	0.893	16.389	0.971	7.992
		RFR	0.865	20.681	0.753	23.433
		MLP	0.688	31.187	0.380	37.112
Rat Skin	sMDC	SVR	0.987	3.814	1.000	0.563
		XGB	0.854	11.650	0.985	3.555
		GBR	0.844	8.110	0.920	8.110
		KNN	0.955	6.578	0.986	3.374
		RFR	0.618	17.265	0.322	23.621
		MLP	−0.053	27.648	0.607	17.985
	mMDC	SVR	0.977	3.210	0.998	0.882
		XGB	0.732	7.853	0.942	4.555
		GBR	0.764	7.996	0.931	4.962
		KNN	0.863	7.403	0.970	3.248
		RFR	0.557	11.823	0.488	13.516
		MLP	0.083	14.820	−0.934	26.268
	LiveBox2	SVR	0.995	2.115	0.997	1.641
		XGB	0.956	6.801	0.983	3.988
		GBR	0.934	7.695	0.942	7.275
		KNN	0.935	7.655	0.978	4.517
		RFR	0.876	10.944	0.854	11.547
		MLP	0.790	10.680	0.994	2.423
Alginate Scaffold	sMDC	SVR	0.982	4.715	0.998	1.516
		XGB	0.901	10.455	0.978	5.530
		GBR	0.934	9.368	0.980	5.367
		KNN	0.928	8.890	0.978	5.529
		RFR	0.727	16.417	0.891	12.397
		MLP	0.097	28.890	0.654	22.119
	mMDC	SVR	0.990	1.952	0.986	1.377
		XGB	0.957	3.956	0.916	3.335
		GBR	0.945	4.618	0.690	6.394
		KNN	0.864	7.405	0.840	4.597
		RFR	0.922	5.594	0.561	7.607
		MLP	0.845	7.200	0.175	10.423
	LiveBox2	SVR	0.939	18.887	0.993	4.586
		XGB	0.530	22.679	0.917	15.599
		GBR	0.816	18.840	0.932	14.138
		KNN	0.884	19.056	0.973	8.918
		RFR	0.440	37.605	0.328	44.417
		MLP	0.194	43.365	−0.866	74.016

**Table 4:** Statistical robustness of test-set model performance across the twelve membrane—device configurations.

Model	Test R^2^, Mean ± SD	Test R^2^, 95% CI	Test RMSE, Mean ± SD	Test RMSE, 95% CI
GBR	0.899 ± 0.105	0.836–0.949	7.535 ± 4.387	5.516–10.249
KNN	0.944 ± 0.074	0.899–0.977	5.237 ± 2.427	4.036–6.641
MLP	−1.144 ± 2.702	−2.777–0.116	33.235 ± 22.621	21.752–45.897
RFR	0.632 ± 0.213	0.514–0.746	17.710 ± 11.777	11.913–24.613
SVR	0.994 ± 0.008	0.989–0.997	1.800 ± 1.258	1.180–2.537
XGB	0.948 ± 0.050	0.918–0.971	6.039 ± 4.218	4.028–8.554

**Table 5:** Cross-validation and training of the SVR model for various microfluidic devices and membrane configurations.

Membrane	Device	Without Augmented Data				With Augmented Data			
		CV R^2^	CV RMSE	Test R^2^	Test RMSE	CV R^2^	CV RMSE	Test R^2^	Test RMSE
PET	sMDC	0.987	4.305	0.999	0.784	0.997	2.248	0.998	2.136
	mMDC	0.981	2.394	0.972	1.373	0.992	6.954	0.994	6.342
	LiveBox2	0.985	4.790	0.999	1.309	0.996	1.959	0.997	2.044
CA	sMDC	0.959	14.786	0.996	3.537	0.993	1.796	0.991	2.255
	mMDC	0.963	1.888	0.995	0.913	0.994	1.022	0.995	0.988
	LiveBox2	0.963	9.066	0.996	3.113	0.996	1.441	0.998	1.386
Rat Skin	sMDC	0.987	3.814	1.000	0.563	0.997	2.341	0.998	2.183
	mMDC	0.977	3.210	0.998	0.882	0.996	3.622	0.997	2.819
	LiveBox2	0.995	2.115	0.997	1.641	0.998	1.552	0.997	1.637
Alginate Scaffold	sMDC	0.982	4.715	0.998	1.516	0.997	2.074	0.997	2.216
	mMDC	0.990	1.952	0.986	1.377	0.996	1.607	0.994	1.888
	LiveBox2	0.939	18.887	0.993	4.586	0.995	4.676	0.997	4.430

**Table 6:** Cubic polynomial correlations derived from SVR model predictive equations for various microfluidic devices and membrane configurations.

Membrane	Device	Cumulative Mass Predictive Equation
PET	sMDC	$cumulative\;mass=-8.75+5.23\times 10^{-1}t+5.04\times 10^{-4}f-1.02\times 10^{-3}t^{2}+9.67\times 10^{-3}tf+1.60\times 10^{-2}f^{2}+1.59\times 10^{-6}t^{3}-8.02\times 10^{-6}t^{2}f-9.28\times 10^{-5}tf^{2}-1.52\times 10^{-4}f^{3}$
	mMDC	$cumulative\;mass=-5.41\times 10^{-1}+1.29\times 10^{-1}t+2.63\times 10^{-5}f-1.39\times 10^{-4}t^{2}+7.99\times 10^{-3}tf+8.32\times 10^{-4}f^{2}+1.50\times 10^{-7}t^{3}-8.97\times 10^{-6}t^{2}f-5.21\times 10^{-5}tf^{2}-8.94\times 10^{-6}f^{3}$
	LiveBox2	$cumulative\;mass=-9.29\times 10^{-1}+8.85\times 10^{-2}t+6.31\times 10^{-5}f-5.46\times 10^{-4}t^{2}+2.37\times 10^{-2}tf+2.00\times 10^{-3}f^{2}-1.25\times 10^{-6}t^{3}-1.45\times 10^{-5}t^{2}f-1.55\times 10^{-4}tf^{2}+1.88\times 10^{-5}f^{3}$
CA	sMDC	$cumulative\;mass=-1.89+1.97\times 10^{-1}t-1.05\times 10^{-4}f-9.85\times 10^{-4}t^{2}+4.49\times 10^{-2}tf-6.50\times 10^{-3}f^{2}+2.17\times 10^{-6}t^{3}-3.55\times 10^{-5}t^{2}f-3.02\times 10^{-4}tf^{2}+4.63\times 10^{-5}f^{3}$
	mMDC	$cumulative\;mass=2.2510^{-1}\times +1.38\times 10^{-1}t-6.83\times 10^{-5}f+2.51\times 10^{-4}t^{2}-8.13\times 10^{-4}tf-2.16\times 10^{-3}f^{2}-2.56\times 10^{-7}t^{3}-4.17\times 10^{-6}t^{2}f-9.67\times 10^{-6}tf^{2}+2.09\times 10^{-5}f^{3}$
	LiveBox2	$cumulative\;mass=2.92-2.03\times 10^{-2}t-7.38\times 10^{-4}f-1.12\times 10^{-3}t^{2}+3.26\times 10^{-2}tf-2.34\times 10^{-2}f^{2}-3.56\times 10^{-6}t^{3}-2.80\times 10^{-5}t^{2}f-2.03\times 10^{-4}tf^{2}+2.43\times 10^{-4}f^{3}$
Rat Skin	sMDC	$cumulative\;mass=7.85+2.94\times 10^{-2}t-1.31\times 10^{-4}f+4.18\times 10^{-4}t^{2}+1.65\times 10^{-2}tf-4.15\times 10^{-3}f^{2}-9.79\times 10^{-7}t^{3}+1.47\times 10^{-7}t^{2}f-1.72\times 10^{-4}tf^{2}+4.15\times 10^{-5}f^{3}$
	mMDC	$cumulative\;mass=-1.49+4.45\times 10^{-2}t+1.86\times 10^{-4}f-1.63\times 10^{-4}t^{2}+9.48\times 10^{-3}tf+5.89\times 10^{-3}f^{2}+2.95\times 10^{-7}t^{3}+6.52\times 10^{-6}t^{2}f-9.95\times 10^{-5}tf^{2}+5.84\times 10^{-5}f^{3}$
	LiveBox2	$cumulative\;mass=4.31-1.53\times 10^{-1}t+3.01\times 10^{-4}f+1.09\times 10^{-3}t^{2}+1.01\times 10^{-2}tf-9.55\times 10^{-3}f^{2}-1.52\times 10^{-6}t^{3}-1.15\times 10^{-5}t^{2}f-3.47\times 10^{-5}tf^{2}+9.11\times 10^{-5}f^{3}$
Alginate Scaffold	sMDC	$cumulative\;mass=-3.34+1.83\times 10^{-1}t+2.70\times 10^{-5}f+1.07\times 10^{-3}t^{2}+8.36\times 10^{-3}tf+8.55\times 10^{-3}f^{2}-1.90\times 10^{-6}t^{3}-2.45\times 10^{-6}t^{2}f-1.09\times 10^{-4}tf^{2}-4.08\times 10^{-6}f^{3}$
	mMDC	$cumulative\;mass=-3.09+9.38\times 10^{-2}t+1.35\times 10^{-4}f+6.07\times 10^{-4}t^{2}+5.98\times 10^{-3}tf+4.28\times 10^{-3}f^{2}-1.29\times 10^{-6}t^{3}-6.04\times 10^{-6}t^{2}f-3.24\times 10^{-5}tf^{2}-4.31\times 10^{-5}f^{3}$
	LiveBox2	$cumulative\;mass=7.73-4.35\times 10^{-1}t-6.82\times 10^{-4}f+2.55\times 10^{-3}t^{2}+4.18\times 10^{-2}tf-2.16\times 10^{-2}f^{2}-5.07\times 10^{-6}t^{3}+1.11\times 10^{-5}t^{2}f-4.01\times 10^{-4}tf^{2}+2.13\times 10^{-4}f^{3}$

**Table 7:** SVR-based steady-state identification and selected operating points across microfluidic devices and membrane configurations.

Membrane	Device	Flow	t_ss_	c_ss_
PET	sMDC	100	178.5	48.24044
	mMDC	100	301.5	57.63312
	LiveBox2	100	223.5	100.641
CA	sMDC	4	9.5	7.985431
	mMDC	100	167.5	20.48226
	LiveBox2	100	145.5	121.16
Rat Skin	sMDC	40	420	185.173
	mMDC	40	420	133.6701
	LiveBox2	100	259.5	99.58941
Alginate Scaffold	sMDC	40	330.5	142.9141
	mMDC	100	305.5	74.58896
	LiveBox2	40	420	447.9636

## Data Availability

The data supporting the findings of this study are available from the corresponding author upon reasonable request.

## Supplementary Materials

The following supporting information can be downloaded at: https://www.mdpi.com/article/10.3390/membranes16070239/s1, Figure S1: Prediction-error plots for PET membrane in (a) sMDC, (b) mMDC, and (c) LiveBox2 devices; Figure S2: Prediction-error plots for CA membrane in (a) sMDC, (b) mMDC, and (c) LiveBox2 devices; Figure S3: Prediction-error plots for rat skin membrane in (a) sMDC, (b) mMDC, and (c) LiveBox2 devices; Figure S4: Prediction-error plots for alginate scaffold membrane in (a) sMDC, (b) mMDC, and (c) LiveBox2 devices; Figure S5: Learning curves for PET membrane in (a) sMDC, (b) mMDC, and (c) LiveBox2 devices; Figure S6: Learning curves for CA membrane in (a) sMDC, (b) mMDC, and (c) LiveBox2 devices; Figure S7: Learning curves for skin rat membrane in (a) sMDC, (b) mMDC, and (c) LiveBox2 devices; Figure S8: Learning curves for alginate scaffold membrane in (a) sMDC, (b) mMDC, and (c) LiveBox2 devices; Table S1: Summary of hyperparameter search spaces used in RandomizedSearchCV; Table S2: Final optimized SVR hyperparameters for each membrane-device configuration; Table S3: Validation of cubic polynomial surrogate equations against SVR predictions.

## Abbreviations

TDDS	transdermal drug delivery systems
AI	artificial intelligence
ML	machine learning
MLP	Multilayer Perceptron
GBR	Gradient Boosting Regressor
XGB	Extreme Gradient Boosting
KNN	K-Nearest Neighbors
RFR	Random Forest Regressor
SVR	Support Vector Regression
sMDC	single-channel microfluidic diffusion chamber
mMDC	multichannel microfluidic diffusion chamber
PET	polyester
CA	cellulose acetate
PPF	peripheral perfusion fluid
CV	cross-validation
RMSE	root mean squared error

## References

[1] Vargason A.M., Anselmo A.C., Mitragotri S. (2021). The evolution of commercial drug delivery technologies. Nat. Biomed. Eng..

[2] Zargar S.M., Kharazi A., Hafshejani D., Eskandarinia A., Rafienia M. (2019). A review of controlled drug delivery systems based on cells and cell membranes. J. Med. Signals Sens..

[3] Lee H., Song C., Baik S., Kim D., Hyeon T., Kim D.H. (2018). Device-assisted transdermal drug delivery. Adv. Drug Deliv. Rev..

[4] Mishra B., Bonde G.V. (2020). Transdermal drug delivery. Controlled Drug Delivery Systems.

[5] Mali A.D. (2015). An updated review on transdermal drug delivery systems. Skin.

[6] Bathe R., Kapoor R. (2015). Transdermal drug delivery system: Formulation, development and evaluation—An overview. Drug Deliv..

[7] Caplin J.D., Granados N.G., James M.R., Montazami R., Hashemi N. (2015). Microfluidic organ-on-a-chip technology for advancement of drug development and toxicology. Adv. Healthc. Mater..

[8] Niculescu A.-G., Chircov C., Bîrcă A.C., Grumezescu A.M. (2021). Fabrication and applications of microfluidic devices: A review. Int. J. Mol. Sci..

[9] Kimura H., Sakai Y., Fujii T. (2018). Organ/body-on-a-chip based on microfluidic technology for drug discovery. Drug Metab. Pharmacokinet..

[10] Van Den Berg A., Mummery C.L., Passier R., Van Der Meer A.D. (2019). Personalised organs-on-chips: Functional testing for precision medicine. Lab A Chip.

[11] Sackmann E.K., Fulton A.L., Beebe D.J. (2014). The present and future role of microfluidics in biomedical research. Nature.

[12] Halldorsson S., Lucumi E., Gómez-Sjöberg R., Fleming R.M.T. (2015). Advantages and challenges of microfluidic cell culture in polydimethylsiloxane devices. Biosens. Bioelectron..

[13] Bhatia S.N., Ingber D.E. (2014). Microfluidic organs-on-chips. Nat. Biotechnol..

[14] Gormley A.J. (2024). Machine learning in drug delivery. J. Control. Release.

[15] Dedeloudi A., Weaver E., Lamprou D.A. (2023). Machine learning in additive manufacturing & Microfluidics for smarter and safer drug delivery systems. Int. J. Pharm..

[16] Hathout R.M. (2021). Machine learning methods in drug delivery. Applications of Artificial Intelligence in Process Systems Engineering.

[17] Kocsis D., Dhinakaran S., Pandey D., Laki A.J., Laki M., Sztankovics D., Lengyel M., Vrábel J., Naszlady M.B., Sebestyén A. (2024). Fluid Dynamics Optimization of Microfluidic Diffusion Systems for Assessment of Transdermal Drug Delivery: An Experimental and Simulation Study. Sci. Pharm..

[18] Luo L., Lane M.E. (2015). Topical and transdermal delivery of caffeine. Int. J. Pharm..

[19] Huang Y., Zhai J., Boukouvala F. (2023). 33rd European Symposium on Computer Aided Process Engineering.

[20] Chan K.Y., Abu-Salih B., Qaddoura R., Al-Zoubi A.M., Palade V., Pham D.-S., Del Ser J., Muhammad K. (2023). Deep neural networks in the cloud: Review, applications, challenges and research directions. Neurocomputing.

[21] Zhang S., Zhu D. (2020). Towards artificial intelligence enabled 6G: State of the art, challenges, and opportunities. Comput. Netw..

[22] Li X.-D., Wang J.-S., Hao W.-K., Wang M., Zhang M. (2022). Multi-layer perceptron classification method of medical data based on biogeography-based optimization algorithm with probability distributions. Appl. Soft Comput..

[23] Torabi T., Bairami A., Ghasemzadeh K., Shojaei M.J., Iulianelli A. (2025). Optimization of sustainable biogas valorization to hydrogen via tri-reforming process in packed bed membrane reactor: An integrated CFD-ML digital twin approach. Renew. Energy.

[24] Heddam S., Kim S., Mehr A.D., Zounemat-Kermani M., Elbeltagi A., Malik A., Kisi O. (2022). A long short-term memory deep learning approach for river water temperature prediction. Current Trends and Advances in Computer-Aided Intelligent Environmental Data Engineering.

[25] Bishnu S.K., Alnouri S.Y., Al Mohannadi D.M. (2025). Stochastic Algorithm-Based Optimization using Artificial Intelligence/Machine Learning Models for Sorption Enhanced Steam Methane Reformer Reactor. Comput. Chem. Eng..

[26] Afzaal H., Farooque A.A., Esau T.J., Schumann A.W., Zaman Q.U., Abbas F., Bos M. (2023). Artificial neural modeling for precision agricultural water management practices. Precision Agriculture.

[27] Khandelwal K., Dalai A.K. (2024). Prediction of individual gas yields of supercritical water gasification of lignocellulosic biomass by machine learning models. Molecules.

[28] Shi R., Xu X., Li J., Li Y. (2021). Prediction and analysis of train arrival delay based on XGBoost and Bayesian optimization. Appl. Soft Comput..

[29] Cunningham P., Cord M., Delany S.J. (2008). Supervised learning. Machine Learning Techniques for Multimedia: Case Studies on Organization and Retrieval.

[30] Zhang Y., Ling C. (2018). A strategy to apply machine learning to small datasets in materials science. npj Comput. Mater..

[31] Vanpoucke D.E., van Knippenberg O.S.J., Hermans K., Bernaerts K.V., Mehrkanoon S. (2020). Small data materials design with machine learning: When the average model knows best. J. Appl. Phys..

[32] Ge J., Yao Z., Wu M., Almeida J.H.S., Jin Y., Sun D. (2025). Tackling data scarcity in machine learning-based CFRP drilling performance prediction through a broad learning system with virtual sample generation (BLS-VSG). Compos. Part B Eng..

[33] Izonin I., Tkachenko R., Berezsky O., Krak I., Kováč M., Fedorchuk M. (2024). Improvement of the ANN-based prediction technology for extremely small biomedical data analysis. Technologies.

[34] Vabalas A., Gowen E., Poliakoff E., Casson A.J. (2019). Machine learning algorithm validation with a limited sample size. PLoS ONE.

[35] Bilali A.E., Taleb A., Bahlaoui M.A., Brouziyne Y. (2021). An integrated approach based on Gaussian noises-based data augmentation method and AdaBoost model to predict faecal coliforms in rivers with small dataset. J. Hydrol..

[36] Smyrnov M., Funcke F., Kabliman E. (2024). Prediction of material toughness using ensemble learning and data augmentation. Philos. Mag. Lett..

[37] Liu D., Kababji S.E., Mitsakakis N., Pilgram L., Walters T., Clemons M., Pond G., El-Hussuna A., Eman K. (2025). Synthetic data generation for augmenting small samples. arXiv.

[38] Ukwuoma C.C., Cai D., Jonathan A.L., Chen N., Sey C., Ntia N.W., Bamisile O., Huang Q. (2024). Enhancing hydrogen production prediction from biomass gasification via data augmentation and explainable AI: A comparative analysis. Int. J. Hydrogen Energy.

[39] Liu Y., Zhu Y., Li D., Huang Z., Bi C. (2023). Computational simulation of mass transfer in membranes using hybrid machine learning models and computational fluid dynamics. Case Stud. Therm. Eng..

[40] Suthaharan S. (2016). Support vector machine. Machine Learning Models and Algorithms for Big Data Classification: Thinking with Examples for Effective Learning.

[41] Basak D., Pal S., Patranabis D.C. (2007). Support vector regression. Neural Inf. Process. -Lett. Rev..

[42] Wang Y., Liao Z., Mathieu S., Bin F., Tu X. (2021). Prediction and evaluation of plasma arc reforming of naphthalene using a hybrid machine learning model. J. Hazard. Mater..

[43] Dhiman H.S., Deb D., Guerrero J.M. (2019). Hybrid machine intelligent SVR variants for wind forecasting and ramp events. Renew. Sustain. Energy Rev..

[44] Ozbas E.E., Aksu D., Ongen A., Aydin M.A., Ozcan H.K. (2019). Hydrogen production via biomass gasification, and modeling by supervised machine learning algorithms. Int. J. Hydrogen Energy.

[45] Chicco D., Warrens M.J., Jurman G. (2021). The coefficient of determination R-squared is more informative than SMAPE, MAE, MAPE, MSE and RMSE in regression analysis evaluation. PeerJ Comput. Sci..

[46] Yaqub M., Lee W. (2022). Modeling nutrient removal by membrane bioreactor at a sewage treatment plant using machine learning models. J. Water Process Eng..

[47] Chang H.-M., Xu Y., Chen S.-S., He Z. (2022). Enhanced understanding of osmotic membrane bioreactors through machine learning modeling of water flux and salinity. Sci. Total Environ..

[48] Khan M.S.H. (2025). Standard deviation. International Encyclopedia of Statistical Science.

[49] DiCiccio T.J., Efron B. (1996). Bootstrap confidence intervals. Stat. Sci..

[50] Zare S., Kargari A. (2022). CFD simulation and optimization of an energy-efficient direct contact membrane distillation (DCMD) desalination system. Chem. Eng. Res. Des..

[51] Myers R.H., Montgomery D.C., Anderson-Cook C.M. (2016). Response Surface Methodology: Process and Product Optimization Using Designed Experiments.

