# Supplementary material for: Machine Learning–Driven Surrogate Modeling and Operating-Point Selection for a Microfluidic Diffusion-Membrane Platform for Transdermal Drug Delivery
Source: Membranes (Basel). 2026 Jul 15;16(7):239. doi: 10.3390/membranes16070239 (PMC13414156; doi:10.3390/membranes16070239)
Supplement: Supplementary file 1 [file membranes-16-00239-s001.zip › membranes-4371715-supplementary.pdf]

2.2. Integration of Machine Learning and Optimization Techniques

Table S1. Summary of hyperparameter search spaces used in RandomizedSearchCV

Model	Hyperparameters sampled	Search range
SVR	Kernel	{RBF, linear}
	Regularization (C)	10^{-2} to 10^{3} (log–spaced)
	Epsilon (ε)	10^{-3} to $10^{-0.3}$ (log–spaced)
	Gamma (ɣ)	{"scale", "auto"}
MLP	Hidden-layer sizes	{(64), (128), (128, 64), (128, 64, 32)}
	Activation	{ReLU, tanh}
	L2 regularization (α)	10^{-6} to 10^{-2} (log–spaced)
	Learning rate	10^{-4} to 10^{-2} (log–spaced)
	Solver	adam
GBR	Number of estimators	100–600
	Learning rate	10^{-3} to $10^{-0.3}$
	Max depth	2–5
	Subsample	{0.6, 0.8, 1.0}
	Min samples leaf	{1, 2, 4, 8}
XGB	Number of estimators	200–800
	Max depth	{3, 4, 5, 6, 8}
	Learning rate	10^{-3} to $10^{-0.3}$
	Subsample	{0.6, 0.8, 1.0}
	Column sample by tree	{0.6, 0.8, 1.0}
	L1 regularization (reg_alpha)	10^{-6} to 10^{-2}
	L2 regularization (reg_lambda)	10^{-3} to 10^{1}
KNN	Number of neighbors	3–51 (odd numbers)
	Weights	{uniform, distance}
	Minkowski p	{1, 2}
	Leaf size	{15, 30, 45, 60}

RFR	Number of estimators	200–700
	Max depth	{None, 6, 8, 12, 16}
	Min samples split	{2, 5, 10}
	Min samples leaf	{1, 2, 4}
	Max features	{"sqrt", "log2", 0.6, 0.8, 1.0}

2.2.1. ML model development

MLP model

The MLP is a commonly utilized architecture within the broader class of artificial neural networks and is particularly effective for both regression and classification problems. It consists of at least three layers, including an input layer, one or more hidden layers, and an output layer. A defining property of MLPs is their fully connected topology, in which every neuron in one layer is linked to all neurons in the next, resulting in a large set of trainable weight parameters. Owing to this flexible structure, MLPs can be adapted to multiple learning paradigms, including supervised, unsupervised, and reinforcement learning [15, 16]. Each neuron in the hidden and output layers applies a non-linear activation function, allowing the network to model complex, high-dimensional relationships present in the data. The overall computation follows a directed acyclic graph, where the transformation from inputs to outputs is governed by the weighted summation of incoming signals, followed by an activation operation. This process is mathematically expressed in Eq. S1:

$$y_j = f(\sum_{i=1}^{n} w_{ij} x_i + b_j) \quad \text{(Eq.S1)}$$

where f is the activation function (e.g., ReLU, sigmoid, tanh), w_{ij} denotes the weight connecting input ito neuron j, x_i represents the input features, b_j is the bias associated with neuron j, and n is the number of features or neurons in the preceding layer [17,18].

In the present study, the hidden layers employed the rectified linear unit (ReLU) activation function [19-22]:

$$\mathrm{ReLU} = \max(0, x) \qquad \text{(Eq.S2)}$$

Model training was performed using the Adam optimization algorithm, with the objective of minimizing the mean squared error (MSE) loss function:

$$L = \frac{1}{N}\sum_{i=1}^{N}(\mathbf{y_i} - \hat{y}_i)^2 \qquad \text{(Eq.S3)}$$

where $y_i$denotes the true values and $\hat{y}_i$the corresponding model predictions.

GBR model

GBR is a powerful ensemble learning methodology that builds predictive models through an iterative, stage-wise optimization process. At each boosting step, the algorithm fits a weak learner—commonly a shallow regression tree—that best approximates the negative gradient of the loss function with respect to the current model predictions. This weak learner, chosen for its ability to most effectively reduce the residual error, is then weighted and incorporated into the ensemble, incrementally improving predictive accuracy [23, 24].

Through this sequential learning mechanism, GBR is capable of capturing complex nonlinear patterns and variable interactions, making it particularly effective for regression tasks characterized by multidimensional and highly coupled dependencies [25, 26].

The resulting model adopts an additive structure expressed as:

$$F_m(x) = \frac{1}{k}\sum_{m=1}^{M}\gamma_m h_m(x) \qquad \text{(Eq.S4)}$$

where $F_M(x)$ denotes the ensemble prediction after M boosting iterations, $h_m(x)$ represents the m-th weak learner (typically a regression tree), and $\gamma_m$is the learning rate that regulates the contribution of each learner to the overall model.

XGB model

XGB is an advanced gradient-boosting algorithm designed to enhance predictive power by learning the relationship between the target variable and a set of input features through sequential model refinement. Within the gradient boosting framework, decision trees are built iteratively, with each new tree minimizing the residual errors left by the existing ensemble. This incremental learning strategy improves model accuracy by repeatedly correcting the shortcomings of previous trees [27, 28].

At each boosting iteration, the model updates predictions according to Eq. S5:

$$\hat{y}_i^{(t+1)} = \hat{y}_i^{(t)} + f_t(x_i) \quad \text{(Eq.S5)}$$

where $\hat{y}_i^{(t)}$ is the prediction from the ensemble up to iteration t, and $f_t(x_i)$ is the output of the newly added tree. This process continues until a predefined number of boosting rounds is reached or a stopping criterion is satisfied [28].

To prevent overfitting, XGB incorporates both pre-pruning (e.g., setting a maximum depth) and post-pruning strategies, which remove branches that do not significantly improve model accuracy. These regularization techniques are especially critical in boosting algorithms, which are prone to overfitting when modeling noisy or high-dimensional datasets. XGB's built-in regularization contributes to its strong generalization performance and its widespread success in real-world regression and classification applications.

The optimization task in XGB seeks to minimize the following objective function [29]:

$$f(L) = \sum_{i=1}^{n} l(y_i, \hat{y}_i) + \sum_{j=1}^{T} \varphi(f_j) \quad \text{(Eq.S6)}$$

where $l(y_i, \hat{y}_i)$ measures the prediction error, and $\phi(f_j)$ is a regularization component penalizing excessive model complexity [28].

The complexity regularization term used in XGB is defined as:

$$\varphi(f) = \gamma T + \frac{1}{2}\lambda \sum_{j=1}^{T} w_j^2 \quad \text{(Eq.S7)}$$

where T is the number of leaf nodes, w_j is the weight associated with leaf j, and γ and λ are regularization parameters that help control overfitting by penalizing deep or overly complex trees [29, 30].

RFR model

The RFR is an ensemble-based ML method that combines the predictions of multiple decision trees to reduce variance and improve overall predictive accuracy [31].

By introducing variability across individual trees—typically through bootstrap sampling and random feature selection—the ensemble gains increased generalization capability. The final prediction for a new input $x$is computed as the average of all tree outputs, following Eq. S8:

$$f(x) = \frac{1}{B}\sum_{b=1}^{B} f_b(x') \quad \text{(Eq.S8)}$$

where $f_b(x')$ denotes the prediction from the b-th tree and Bis the total number of trees in the forest [16].

KNN model

KNN algorithm is a simple yet highly adaptable ML method widely used for both regression and classification tasks. Its predictive strategy is based on the assumption that samples located close to one another in the feature space are likely to exhibit similar outputs. For a given query point, the algorithm identifies its k nearest neighbors and derives the prediction from their corresponding target values.

Distance or similarity between the query point and training samples can be computed using various metrics, with Euclidean, Manhattan, and Minkowski distances being the most common choices. In this study, distances between samples were computed using the Minkowski metric, which generalizes both the Manhattan and Euclidean distance measures. For two samples $x = (x_1, x_2, \dots, x_n)$ and $y = (y_1, y_2, \dots, y_n)$ in an n-dimensional feature space, the Minkowski distance of order p is defined as Eq. S9 [32, 33].

$$d(x, y) = (\sum_{i=1}^{n} |x_i - y_i|^p)^{1/p} \quad \text{(Eq.S9)}$$

Where x and y represent the feature vectors of the two points being compared, and p controls the form of the distance function. When $p = 1$, the metric reduces to the Manhattan distance; when $p = 2$, it becomes the Euclidean distance; and higher values of p increasingly emphasize larger feature differences.

In KNN regression, the predicted output $\hat{y}_q$ is obtained by averaging the target values of the k closest neighbors [34]. The prediction rule for KNN regression follows:

$$\hat{y}_q = \frac{1}{k} \sum_{i \in \mathcal{N}_k(x_q)} y_i \quad \text{(Eq.S10)}$$

where $N_k(x_q)$ denotes the set of indices of the knearest neighbors to sample x_q, and $y_i$corresponds to the target values associated with those neighbors [35].

3. Results and Discussion

3.2. Performance evaluation of SVR model

Table S2. Final optimized SVR hyperparameters for each membrane-device configuration

Membrane	Device	Kernel	C	Epsilon	Gamma
	sMDC	RBF	1000.0000	0.0010	auto
**PET**	mMDC	RBF	0.6579	0.0079	scale
	LiveBox2	RBF	351.1192	0.0079	auto
	sMDC	RBF	123.2847	0.0316	scale
**CA**	mMDC	RBF	0.6579	0.0079	scale
	LiveBox2	RBF	1000.0000	0.0010	auto
	sMDC	RBF	351.1192	0.0079	auto
**Rat Skin**	mMDC	RBF	1000.0000	0.0010	auto
	LiveBox2	RBF	1000.0000	0.0010	auto
	sMDC	RBF	0.6579	0.0079	scale
**Alginate Scaffold**	mMDC	RBF	0.6579	0.0079	scale
	LiveBox2	RBF	351.1192	0.0079	auto

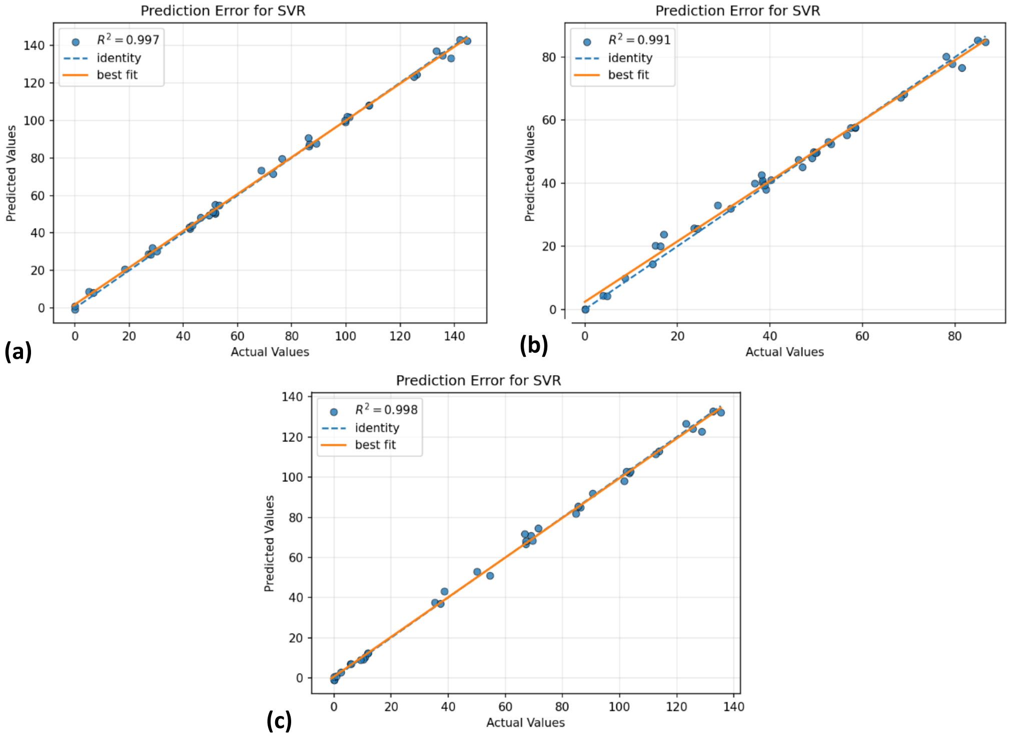

Figure S1. Prediction-error plots for PET membrane in (a) sMDC, (b) mMDC, and (c) LiveBox2 devices

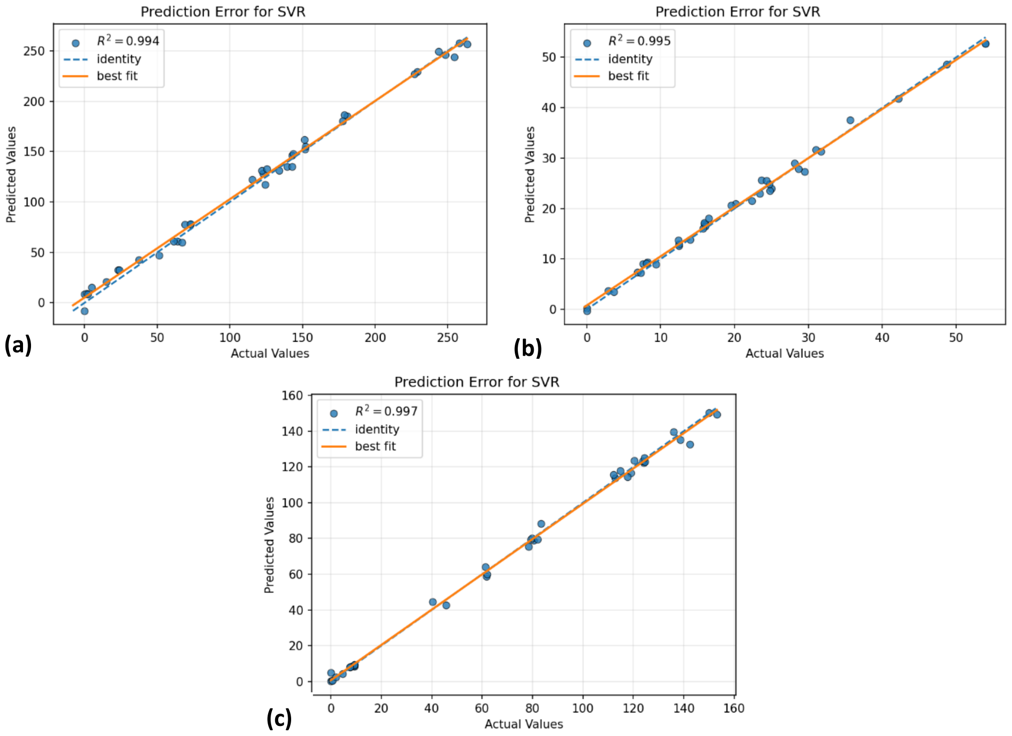

Figure S2. Prediction-error plots for CA membrane in (a) sMDC, (b) mMDC, and (c) LiveBox2 devices

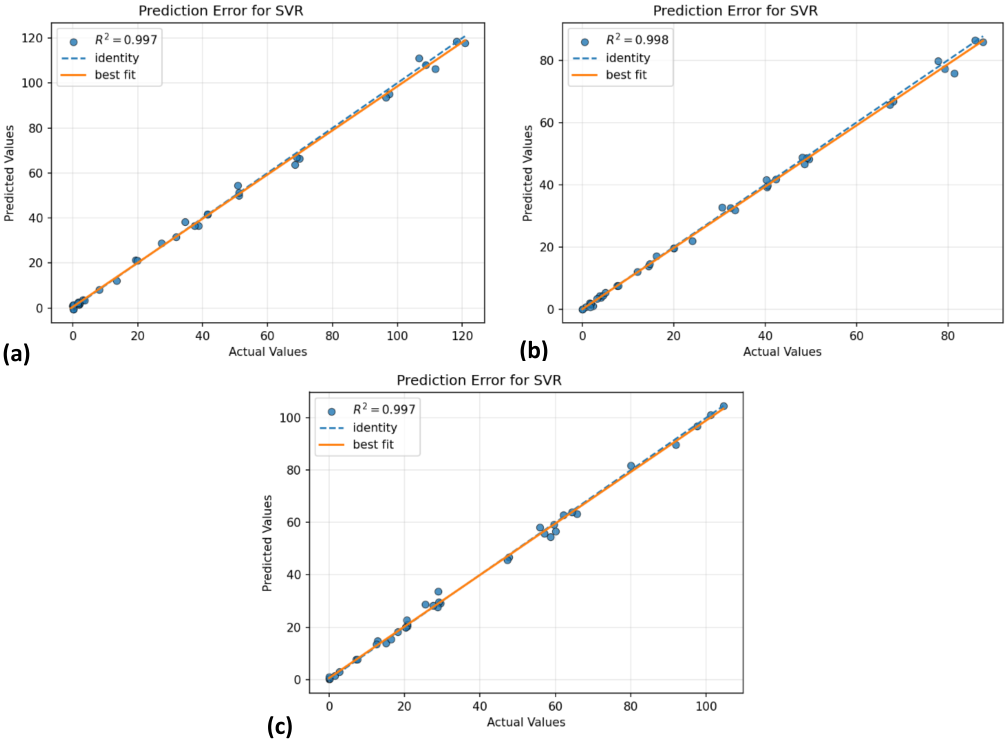

Figure S3. Prediction-error plots for rat skin membrane in (a) sMDC, (b) mMDC, and (c) LiveBox2 devices

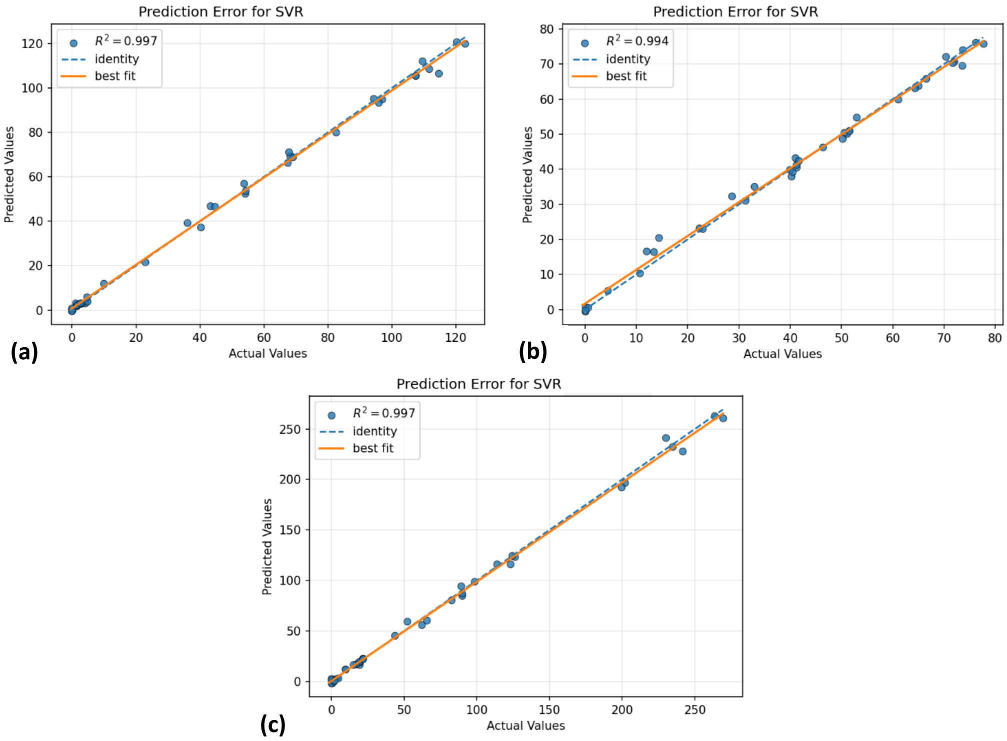

Figure S4. Prediction-error plots for alginate scaffold membrane in (a) sMDC, (b) mMDC, and (c) LiveBox2 devices

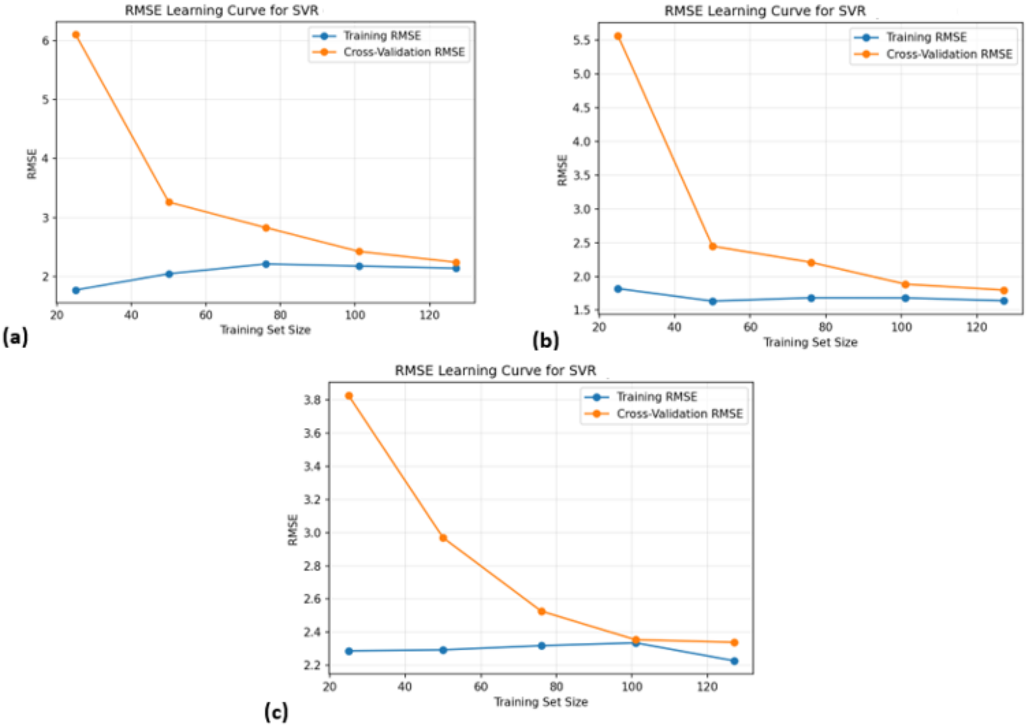

Figure S5. Learning curves for PET membrane in (a) sMDC, (b) mMDC, and (c) LiveBox2 devices.

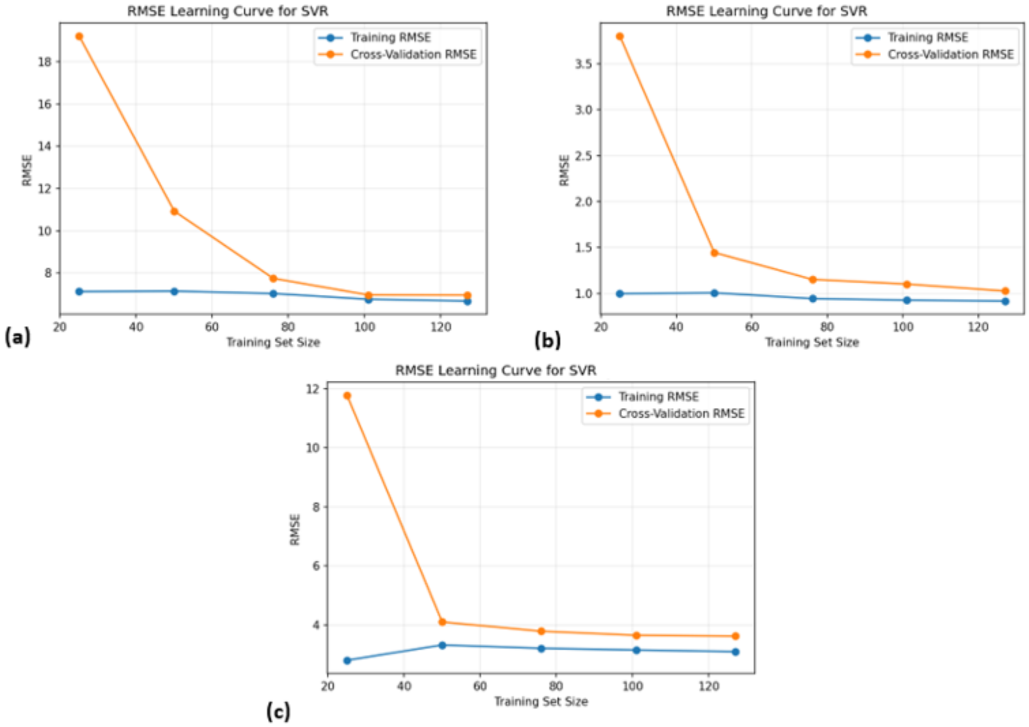

Figure S6. Learning curves for CA membrane in (a) sMDC, (b) mMDC, and (c) LiveBox2 devices.

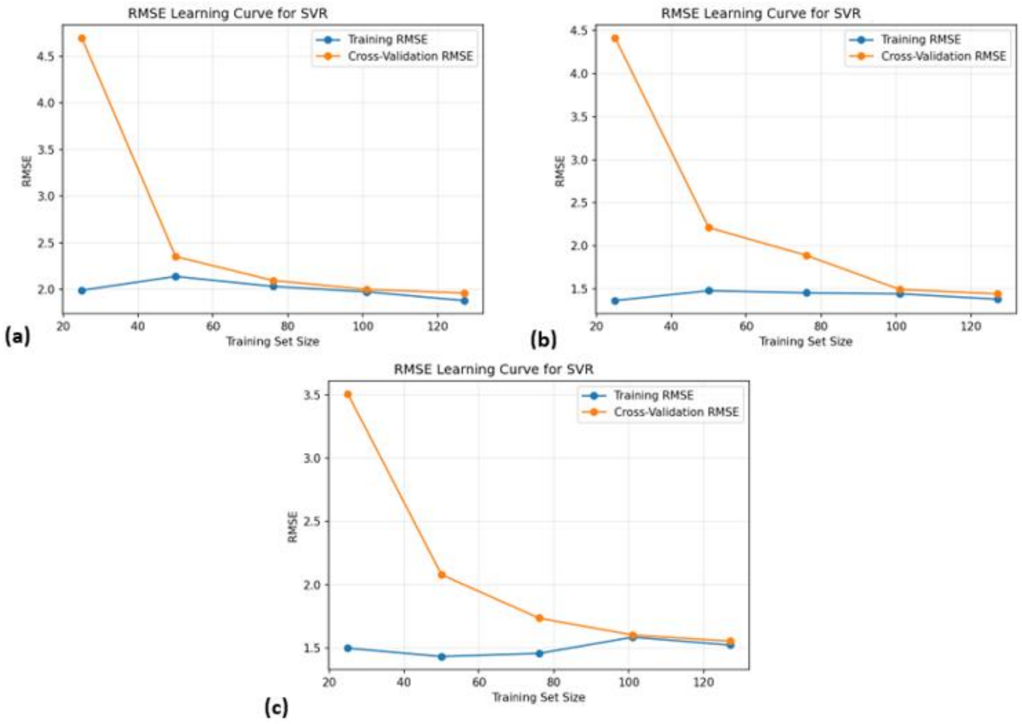

Figure S7. Learning curves for skin rat membrane in (a) sMDC, (b) mMDC, and (c) LiveBox2 de-vices.

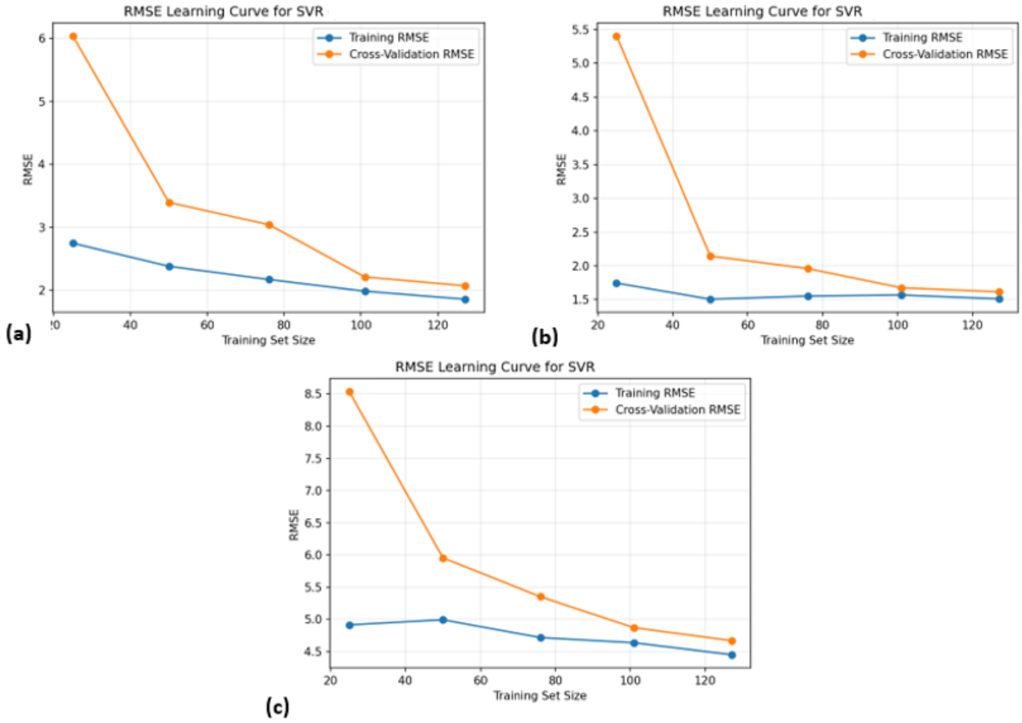

Figure S8. Learning curves for alginate scaffold membrane in (a) sMDC, (b) mMDC, and (c) LiveBox2 devices.

The cubic polynomial equations were validated by comparing their predicted outputs with the corresponding SVR-predicted values for each membrane-device configuration. This analysis was performed to determine whether the analytical equations faithfully reproduced the SVR surrogate model. As shown in Table S3, the polynomial equations showed excellent agreement with the SVR predictions, with R^2 values ranging from 0.9844 to 0.9996 on the test dataset. The mean R^2 values were 0.9957 ± 0.0040 for the training dataset and 0.9950 ± 0.0048 for the test dataset. These results confirm that the third-degree polynomial equations accurately approximate the SVR-generated permeation profiles and can therefore be used as simplified analytical representations of the trained SVR surrogate models.

Table S3. Validation of cubic polynomial surrogate equations against SVR predictions

**Design**	**R^2 vs SVR, train**	**R^2 vs SVR, test**
1A	0.9943	0.9944
1B	0.9983	0.9978
1C	0.9996	0.9996
2A	0.9877	0.9872
2B	0.9948	0.9948
2C	0.9882	0.9844
3A	0.9993	0.9995
3B	0.9974	0.9979
3C	0.9951	0.9940
4A	0.9984	0.9976
4B	0.9971	0.9939
4C	0.9982	0.9987
**Mean ± SD**	**0.9957 ± 0.0040**	**0.9950 ± 0.0048**
